# Temporal and spatial biosonar activity of the recently established uppermost Yangtze finless porpoise population downstream of the Gezhouba Dam: Correlation with hydropower cascade development, shipping, hydrological regime, and light intensity

**DOI:** 10.1002/ece3.11346

**Published:** 2024-05-06

**Authors:** Zhi‐Tao Wang, Peng‐Xiang Duan, Tomonari Akamatsu, Ke‐Xiong Wang, Ding Wang

**Affiliations:** ^1^ School of Marine Science Ningbo University Ningbo China; ^2^ Institute of Hydrobiology Chinese Academy of Sciences Wuhan China; ^3^ Ocean Policy Research Institute The Sasakawa Peace Foundation Tokyo Japan

**Keywords:** boat traffic, Gezhouba dam, hydrological regime, hydropower cascade development, light intensity, Yangtze finless porpoises

## Abstract

Numerous dams disrupt freshwater animals. The uppermost population of the critically endangered Yangtze finless porpoise has been newly formed below the Gezhouba Dam, however, information regarding the local porpoise is scarce. Passive acoustic monitoring was used to detect the behaviors of porpoises below the Gezhouba Dam. The influence of shipping, pandemic lockdown, hydrological regime, and light intensity on the biosonar activity of dolphins was also examined using Generalized linear models. Over the course of 4 years (2019–2022), approximately 848, 596, and 676 effective monitoring days were investigated at the three sites, from upstream to downstream. Observations revealed significant spatio‐temporal biosonar activity. Proportion of days that are porpoise positive were 73%, 54%, and 61%, while porpoise buzz signals accounted for 78.49%, 62.35%, and 81.30% of all porpoise biosonar at the three stations. The biosonar activity of porpoises was much higher at the confluence area, particularly at the MZ site, during the absence of boat traffic, and during the Pandemic shutdown. Temporal trends of monthly, seasonal, and yearly variation were also visible, with the highest number of porpoises biosonar detected in the summer season and in 2020. Significant correlations also exist between the hydrological regime and light intensity and porpoise activity, with much higher detections during nighttime and full moon periods. Hydropower cascade development, establishment of a natural reserve, fish release initiatives, and implementation of fishing restrictions may facilitate the proliferation of the porpoise population downstream of the Gezhouba Dam within the Yichang section of the Yangtze River. Prioritizing restoration designs that match natural flow regimes, optimize boat traffic, and reduce noise pollution is crucial for promoting the conservation of the local porpoises.

## Graphical abstract

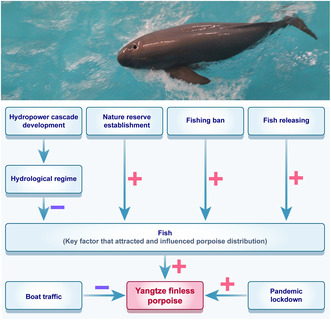

## 1 INTRODUCTION

River ecosystems are rich in biodiversity, yet dams are harming river systems worldwide (Palmer & Ruhi, 2019; Wang et al., 2016). Globally, there were 58,713 major dams as of April 2020, using the criteria of the International Commission On Large Dams (Icold, 2023). Over 50 percent of the world's major river systems are influenced by dams (Nilsson et al., 2005). In addition to their functions in flood control, renewable energy generation, and navigation, dams can affect the seasonal dynamics of river hydrology that structure river environments both upstream and downstream, which can have devastating effects on species that rely on freshwater habitats (Brownell et al., 2017; Ezcurra et al., 2017).

The basin area of the Yangtze River in China is one of the largest in the world, yet hydropower cascade growth on the river is reaching extremes. By the end of the year 2000, 45,694 dams had been constructed on the Yangtze River, according to incomplete records (Li, Zou, et al., 2007). Among the numerous dams, the hydroelectric cascade development of the Gezhouba Dam and the Three Gorges Dam, both located in Yichang, Hubei Province, Central China, stand out. The Gezhouba Dam was the first large‐scale hydroelectric facility on the Yangtze River and the largest low‐head, large‐flow, run‐of‐river hydroelectric facility in the world. The Three Gorges Dam, located 38 km upstream from the Gezhouba Dam, was the largest hydroelectric power station in the world (Wu et al., 2003).

The Gezhouba Dam is the dividing line between the Yangtze's upper and middle reaches. After its impoundment in 1981, the Yangtze finless porpoise (*Neophocaena asiaeorientalis asiaeorientalis*) was unable to migrate upstream of the Yangtze River. The Yangtze finless porpoise is the only freshwater population of porpoises in the world and is currently confined to the middle and lower reaches of the Yangtze River's main stem, as well as Poyang Lake and Dongting Lake (Wang, 2009). It is designated as Critically Endangered on the International Union for Conservation of Nature Red List (Wang et al., 2013) and Appendix I of the Convention on International Trade in Endangered Species of Wild Fauna and Flora (CITES) (https://cites.org/sites/default/files/eng/app/2020/E‐Appendices‐2020‐08‐28.pdf). Since 2021, it has been recognized as a first‐class protected animal in China.

During the historical study of cetaceans in the Yangtze River, the uppermost sighting of a finless porpoise occurred in 2006, roughly 170 km downstream of the Gezhouba Dam in Zhichen (Zhao et al., 2008), approximately 10 km downstream of the Gezhouba Dam at Yanzhiba in 2012 (Mei et al., 2014) and just below the Gezhouba Dam in 2017 (Huang et al., 2020). These upper limits of sightings are each closer to the dam than the earlier sighting. Yangtze finless porpoises have been often sighted in Yanzhiba since 2008 and below the Gezhouba Dam since 2016 (He GW, personal communication) and have recently developed the uppermost porpoise population in the Yangtze River.

Understanding the occurrence and habitat preferences of the finless porpoise is crucial for the conservation of local populations. The finless porpoise population in the Yichang region below the Gezhouba Dam in the Yangtze River has been investigated less than other groups. The finless porpoise, known for its swift surface breaths and lack of a dorsal fin, ranks among the most challenging cetacean species to observe visually. Cetaceans' high level of vocalization facilitates their acoustic surveillance (Würsig, 1989). On average, Yangtze finless porpoises emitted narrow‐band high‐frequency sonar click trains every 6.4 s (Akamatsu et al., 2007; Würsig, 1989), facilitating their monitoring through passive acoustic methods. Passive acoustic monitoring (PAM) is an noninvasive and reliable method to investigate acoustically active animals and provide information on their activities and distributions at high spatial and temporal resolutions (Wang et al., 2022; Zimmer, 2011). PAM has been extensively employed in studying the distribution and habitat preferences of the Yangtze finless porpoise (Wang, Akamatsu, et al., 2015; Wang et al., 2014, 2024).

We postulated that a resident porpoise population has established itself downstream of the Gezhouba Dam within the Yichang section of the Yangtze River. In order to stop the spread of COVID‐19 at the start of the year 2020, lockdowns were implemented globally, drastically restricting human mobility. This involved the temporary halt of all non‐essential travel, including boat traffic and transportation through bridges. These measures collectively resulted in a significant reduction in anthropogenic noise pollution, notably attributed to boat traffic (Lecocq et al., 2020). The navigation of vessels, alongside its potential negative effects on porpoises, was constrained. We further hypothesis that the formation of this porpoise population is attributed to the synergistic impact of anthropogenic activities, namely hydropower cascade development, fishery activity management and pandemic lockdown, as well as local environmental factors such as hydrological conditions. This study aimed to characterize the biosonar activity of the Yangtze finless porpoise downstream of the Gezhouba Dam using PAM. In addition, factors, such as the anthropogenic activities of boat traffic, pandemic lockdown, and hydropower cascade development, as well as the environmental factors of water level, water flux, and light intensity, were investigated for their potential effects on the temporal presence of this indigenous species below the Gezhouba Dam of the Yangtze River.

## 2 MATERIALS AND METHODS

**FIGURE 1:**
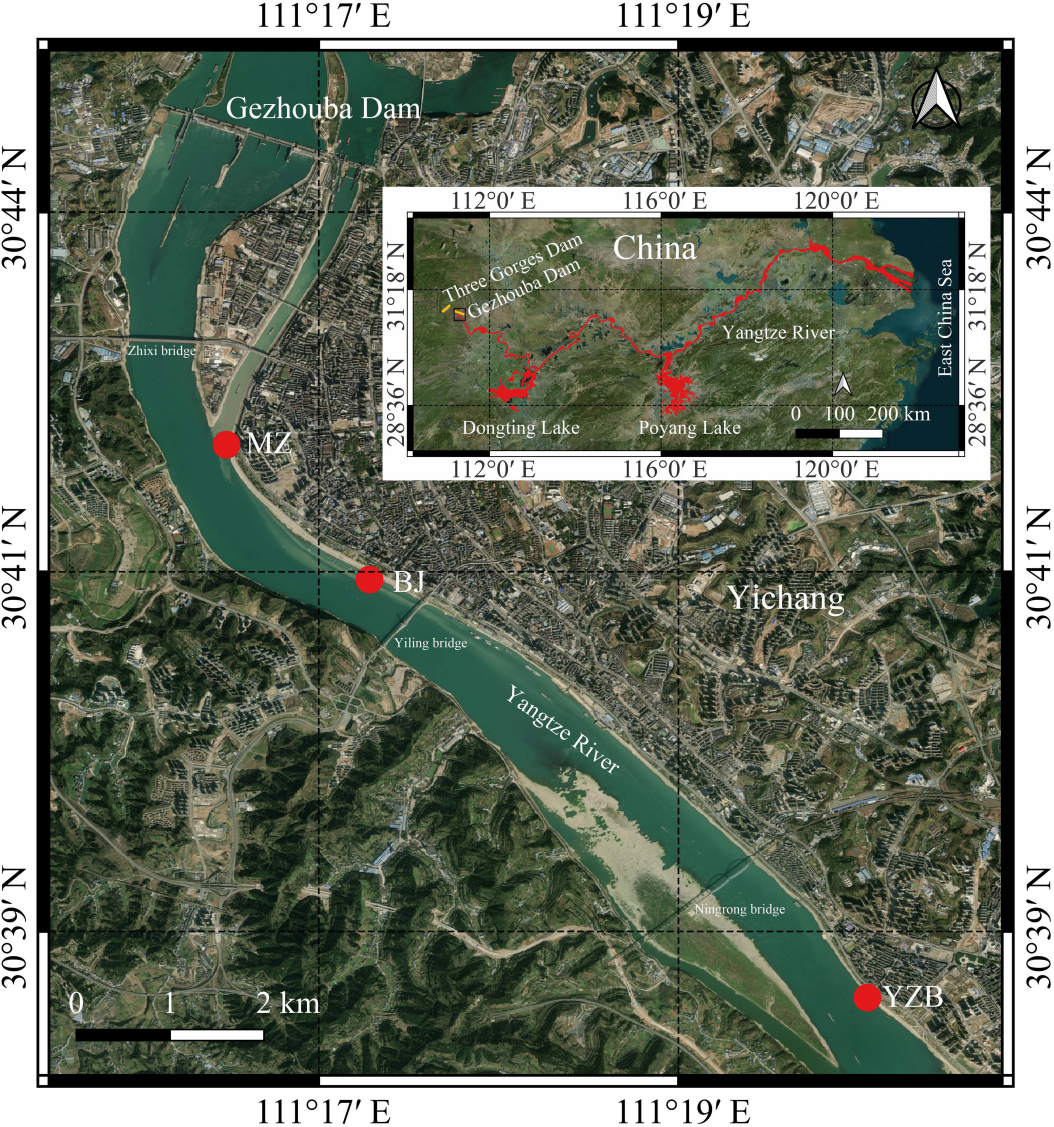
Locations where static acoustic monitoring was conducted downstream of the Gezhouba Dam in the Yangtze River. The red‐shaded area in the inset refers to the Yangtze finless porpoise's distribution area in the middle and lower mainstream of the Yangtze River and Poyang and Dongting Lakes, and the black box marks the area of the Yangtze River downstream of Gezhouba Dam in Yichang city. On the basis of Google Maps, a map was made with QGIS software.

At three locations downstream of the Gezhouba Dam within the Chinese Sturgeon Nature Reserve of the Yangtze River in Yichang, Hubei Province, passive acoustic monitoring was done. Miaozui marine affairs wharf (30°42′15″ N, 111°16′11″ E, hereafter short for MZ) is positioned 3.5 km downstream of the Gezhouba Dam and serves as the most upstream static monitoring station (Figure 1). The MZ site was situated at a confluence area between two tributaries of the Yangtze River (Figure 1). The intermediate static monitoring site was located 5.9 km downstream of the Gezhouba Dam at the Binjiang fisheries administration law enforcement wharf (30°41′21″ N, 111°17′8″ E, hereafter short for BJ) (Figure 1). The lower static monitoring site was located 13 km downstream of the Gezhouba Dam at the Yanzhiba canal administration wharf (30°38′33″ N, 111°20′27″ E, hereafter short for YZB) (Figure 1). Both the BJ and YZB sites were situated within the main channel of the Yangtze River (Figure 1). The choice of these locations was influenced by factors including historical records of porpoise sightings, accessibility to logistical support, and considerations for equipment safety.

### 2.1 Acoustic data collection

This study uses F‐POD (Chelonia Limited, UK), an autonomous acoustic data loggers to monitoring the porpoise. The F‐POD hydrophone is comparable to the B&K 8103 hydrophone in that it is omnidirectional in the frequency range of 20 to 160 kHz and is capable of recording the biosonar signals of echo‐locating cetaceans other than sperm whales. The detection range of cetaceans is affected by ambient noise, especially that produced by boat traffic. Porpoises, for instance, have an estimated maximum detection range of approximately 400 m (https://www.chelonia.co.uk/fpod_home_page.htm). A trained detector onboard picks train clicks and signal were sampled at a sample rate of 1 MHz and then up‐sampled to 4 million samples per second (to 250 ns time resolution). With more precise peak placements, the up‐sampling procedure can correct the wave number of the loudest cycle and the number of amplitude inflections. Twelve characteristics, including duration, number of cycles, subsequent wavelengths and amplitudes around the click's loudest cycle (five wave periods and three amplitudes), number of amplitude inflections, period range, and time of the click, were recorded (https://www.chelonia.co.uk/fpod_home_page.htm).

The hydrophone of the F‐POD was deployed roughly 2 m below the water's surface. The water depths at the acoustic monitoring sites of MZ, BJ, and YZB were 22, 17, and 24 m, respectively. The average diving depth of finless porpoises in an oxbow of the Yangtze River ranged from 2.56 to 3.41 m, with a maximum diving depth recorded at 20.42 m (though this may be constrained by the depth of the water in the oxbow) (Akamatsu et al., 2002). Thus, the monitoring locations' depths fall within the activity range of the porpoises. Each F‐POD was recovered from the field every 1–4 months for data download, with the maximum running time being roughly 140 days. For the examination of the temporal pattern of porpoise biosonar activity, days without 24 h of continuous recording were omitted, particularly during data logger deployment and retrieval.

### 2.2 Acoustic data analysis

F‐POD data were processed with the software F‐POD.exe (Chelonia Limited, Mousehole, Cornwall, UK), and KERNO‐F classifies extracted coherent click trains. In four phases, the KERNO‐F classifier conducted offline detection and classification of click trains based on the 12 characteristics of clicks recorded by the F‐POD. First, trains of comparable, regularly spaced clicks were found. Second, the coherence of the train's subsequent clicks and intervals was evaluated. Thirdly, a threshold criterion for train coherence was applied, classifying each train's ‘quality’ as either good, moderate, low, or questionable. Fourthly, each train was classified into one of the four categories (narrow‐band high‐frequency clicks (signals resembling clicks from porpoise species, such as harbor porpoises and finless porpoises), broadband high‐frequency clicks (signals resembling clicks from dolphin species), boat sonar, and unclassified signal category). In order to prevent a portion of the dataset from yielding different findings if processed separately, click train identification and classification were undertaken within single clock minutes and not across minute boundaries.

During feeding, cetaceans often emit rapid echolocation clicks known as Buzz signals, characterized by a minimum inter‐click interval of less than 10 ms (Todd et al., 2009; Wang, Akamatsu, et al., 2015; Wang et al., 2014). Buzz signal have been frequently utilized in cetacean research as an indicator of feeding activity (Todd et al., 2009; Wang, Akamatsu, et al., 2015; Wang et al., 2014). Finless porpoises also emit buzz signals during prey capture (Akamatsu et al., 2010; Wang, Akamatsu, et al., 2015; Wang et al., 2014). This study employed buzz signals to infer patterns of porpoise feeding behavior. Narrow‐band high‐frequency clicks with a minimum inter‐click interval of less than 10 ms were further extracted and labeled as buzz signals. Sonar activity of porpoises was exported as porpoise click detection positive rate per minute (DPRM), the number of porpoise click trains per minute, buzzes DPRM, and number of buzzes per minute.

### 2.3 Anthropogenic and environmental data

The impact of anthropogenic activities, hydrological regime, and light intensity on the temporal patterns of porpoise sonar activity was examined using gathered parameters. Human activity included the COVID‐19 pandemic lockdown and boat traffic. The hydrological regimes of water level and water flux have been gathered. Light intensity included both the diurnal and lunar cycles. From F‐POD data, boat traffic conditions were also extracted as boat sonar DPRM and the number of boat sonar detections per minute. Due to the COVID 19 pandemic, Yichang was locked down continuously from 24th January to 15th March 2020. Data collected during this period were compared to data collected during non‐lockdown periods to determine the influence of lockdown on porpoise sonar activity. Using custom‐made Python software, the hydrological regime of the water level and water flow at the acoustic monitoring location was collected from the official database of the water resources database (https://slt.hubei.gov.cn/sjfb/). In order to study diel patterns, each day was divided into three light periods: darkness, twilight, and day (Duan et al., 2023; Narganes Homfeldt et al., 2022). In order to investigate lunar trends, nighttime was separated into three periods: the new moon, the quarter moon, and the full moon (Wang, Nachtigall, et al., 2015). The date was split into spring, summer, autumn and winter according to the local city's astronomical seasons (https://www.timeanddate.com/calendar/seasons.html) to explore seasonal patterns.

### 2.4 Statistical analysis

The association of the temporal pattern of porpoise biosonar parameters with boat traffic, water level, water flux, and light intensity was analyzed using a generalized linear model (GLM). Due to the observation of collinearity between water level and water flux (paired sample correlations coefficient = .996, *p* < .001), either water flux or water level was incorporated into the GLM model. Incorporating the porpoise biosonar parameters as dependent variables, site, boat traffic, diel, month, season, and year as fixed factors, and water flux as covariates, a seven‐way ANOVA (ANalysis Of VAriance) complete factorial design was done for the combined data from the three sites. Six‐way ANOVA Full factorial design was implemented at each site by using porpoise biosonar characteristics as dependent variables, as well as boat traffic, diel, month, season, and year as fixed factors, and water flux as covariates in the model. For lunar pattern analysis, nighttime subset data were collected and analyzed using a seven‐way ANOVA on the pooled database, with porpoise biosonar parameters as dependent variables, site, boat traffic, lunar, month, season, and year as fixed factors, and water flux as a covariate. Six‐way ANOVA Full factorial design was implemented for each site during nighttime by incorporating porpoise biosonar measurements as dependent variables, as well as boat traffic, lunar, month, season, and year as fixed factors, and water flow as covariates. In the GLM model, interaction variables between or among fixed factors were also incorporated. Fixed variables and interaction terms with *p*‐values below a predetermined threshold were omitted from the final model. When significant differences were identified for either fixed factor, post hoc multiple comparison tests were undertaken using Tamhane's T2 approach (variances not equal by Levene statistic, *p* < .05) or Tukey's Honestly Significant Difference (HSD) method. Spearman's rho approach calculated the correlation between porpoise biosonar detections and boat traffic. During the pandemic lockdown time, only the MZ site had a recorder in operation, and the Mann–Whitney *U*‐test was used to determine the impact of lockdown on porpoise sonar activity by comparing data obtained during the lockdown period to that of the non‐lockdown period.

Data analysis was furnished using SPSS software version 26.0 (IBM Corp. IBM SPSS Statistics for Windows, Armonk, NY, USA) and a custom‐made script based on Matlab R2018b (The Mathworks, Natick, MA, USA).

## 3 RESULTS

**FIGURE 2:**
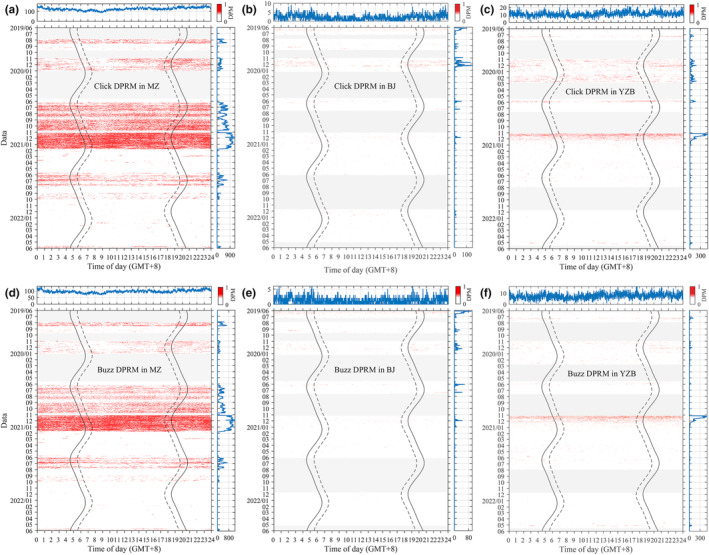
Porpoise click detection positive rate per minute (DPRM) in MZ (a), BJ (b) and YZB (c) and corresponding porpoise buzz DPRM (d–f) as a function of time of day (*x*‐axis) and date (*y*‐axis). Results are provided for every minute. The light gray horizontal region indicates days without a full 24 h recording. The solid black lines indicate the start of dawn and the end of dusk. The dashed broken lines represent the end of dawn and the start of dusk. Line plots on the top denote the diel pattern of either porpoise DPRM or porpoise buzz DPRM, respectively, over the investigation period. The line plot on the right side represents either the summed porpoise click DPRM or the porpoise buzz DPRM daily.

**FIGURE 3:**
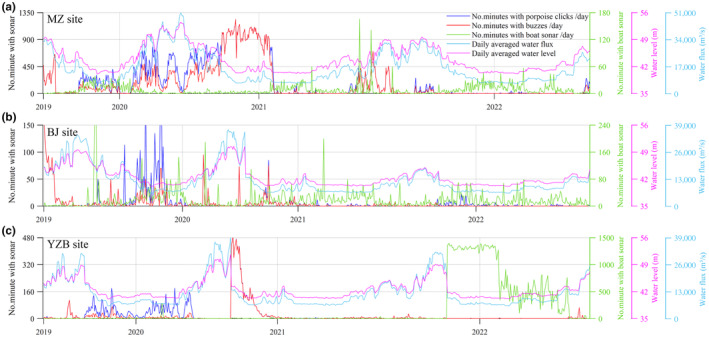
Line plot of the number of minutes with porpoise clicks, porpoise buzz and boat in each day superimposed with the daily averaged water flux and water level in MZ (a), BJ (b) and YZB (c). Each day's results were displayed except days without a full 24 h recording. For clarity, the maximum scale of the left y‐axis varies among the three figures.

All three sites underwent static acoustic monitoring from 2019 to 2022, totaling 2120 effective monitoring days (days with 24 h of full‐time recording or a cumulative data gap of fewer than 2 h) (Table 1, Figures 2 and 3). Eleven rounds of field equipment collection and release were done at the MZ site during the course of 848 monitoring days. At both the BJ and YZB sites, nine rounds of equipment collection and release were done, with a total duration of 596 and 676 effective monitoring days, respectively (Table 1). MZ, BJ, and YZB recorded porpoise biosonar signals on 615, 323, and 414 days, respectively. Proportion of days that are porpoise positive was 73%, 54%, and 61% for MZ, BJ, and YZB, respectively and 64% for the combined data from the three sites (Table 1).

**TABLE 1:** Results of porpoise biosonar and boat sonar detection.

Sites	Deploy date	Retrieve date	Analyzed days	No. days with porpoise sonar (positive rate)	No. porpoise click DPM	No. porpoise click trains	No. buzz DPM	No. buzzes	No. boat sonar DPM	No. boat sonars
MZ	07/28/2019	09/22/2019	55	54 (98%)	6721	54,519	6702	53,679	82	119
	10/29/2019	01/02/2020	64	64 (100%)	12,784	85,784	5450	19,725	1093	3452
	05/18/2020	07/20/2020	63	56 (89%)	19,390	106,050	10,526	32,208	419	1594
	07/21/2020	10/23/2020	94	94 (100%)	45,831	258,883	25,995	82,524	134	321
	11/04/2020	01/17/2021	75	75 (100%)	72,069	1,164,641	71,971	1,132,645	170	430
	01/18/2021	03/23/2021	65	52 (80%)	4633	47,623	2986	20,665	855	2893
	03/24/2021	06/04/2021	73	33 (45%)	1789	7087	853	2309	451	1479
	06/05/2021	07/28/2021	54	54 (100%)	11,645	82,841	11,562	81,404	600	1826
	07/29/2021	11/20/2021	115	50 (43%)	2099	9080	1203	3632	408	1147
	11/21/2021	01/24/2022	65	30 (46%)	88	303	29	67	1126	3764
	01/25/2022	05/30/2022	125	53 (42%)	1767	6422	910	2140	993	3440
	Sum		848	615 (73%)	178,816	1,823,233	138,187	1,430,998	6331	20,465
BJ	06/04/2019	07/07/2019	32	31 (97%)	746	2254	742	2227	58	143
	07/26/2019	09/21/2019	56	40 (71%)	177	896	129	725	1304	4728
	10/29/2019	01/07/2020	69	56 (81%)	1464	4071	401	851	2277	7359
	05/16/2020	07/19/2020	63	34 (54%)	200	933	199	923	479	1277
	11/04/2020	01/18/2021	75	60 (80%)	270	1004	247	953	1398	4284
	01/19/2021	03/23/2021	64	17 (27%)	32	63	8	19	1801	5647
	03/24/2021	06/04/2021	72	25 (35%)	56	450	51	444	615	1315
	11/21/2021	01/25/2022	65	43 (66%)	126	206	11	35	1364	8184
	01/26/2022	05/06/2022	100	17 (17%)	22	41	1	7	1454	5104
	Sum		596	323 (54%)	3093	9918	1789	6184	10,750	38,041
YZB	06/06/2019	07/28/2019	51	41 (80%)	524	2645	509	2598	11	34
	10/29/2019	02/24/2020	117	117 (100%)	5459	10,796	1105	1881	999	6968
	05/16/2020	07/20/2020	64	24 (38%)	1050	2039	177	327	104	226
	11/04/2020	01/18/2021	75	73 (97%)	8326	42,943	8265	42,173	236	483
	01/19/2021	03/23/2021	64	42 (66%)	67	75	9	13	254	942
	03/24/2021	06/05/2021	74	61 (82%)	251	1223	248	1213	127	415
	06/06/2021	07/30/2021	54	33 (61%)	156	536	154	534	47	90
	11/21/2021	01/25/2022	65	2 (3%)	5	8	5	8	84,703	262,288
	01/26/2022	05/17/2022	112	21 (19%)	185	1467	184	1443	38,449	176,518
	Sum		676	414 (61%)	16,023	61,732	10,656	50,190	124,931	447,967

*Note*: Positive rate was obtained by dividing the number of porpoise click‐positive days by the whole analyzed days.

Abbreviation: DPM, detection positive minute.

Out of the 1,221,120 min evaluated at the MZ site, 178,816 min comprise porpoise clicks, and 138,187 min contain buzz signals, accounting for 14.64% and 11.32%, respectively. There were a total of 1,823,233 click trains and 1,430,998 buzzes identified. Porpoise buzz signals account for 78.49% of all the porpoise click trains (Table 1).

Of the 858,240 min evaluated at the BJ site, 3093 min have porpoise clicks, and 1789 min contain porpoise buzz signals, representing 0.36% and 0.21%, respectively (Table 1). There was a total of 9918 click trains and 6184 buzzes reported from porpoises. 62.35% of porpoise click trains are comprised of porpoise buzz signals (Table 1).

In the YZB site, of all the analyzed, 973,440 and 16,023 min contain porpoise clicks, and 10,656 min contain buzz signals, which account for 1.65% and 1.09%, respectively (Table 1). There was a total of 61,732 click trains, and 50,190 porpoise buzzes observed. Porpoise buzz signals represent 81.30% of all porpoise click trains (Table 1). Porpoise buzz signals make up 78.49% of all porpoise click trains based on the combined data from the three sites.

### 3.1 Spatial pattern

**FIGURE 4:**
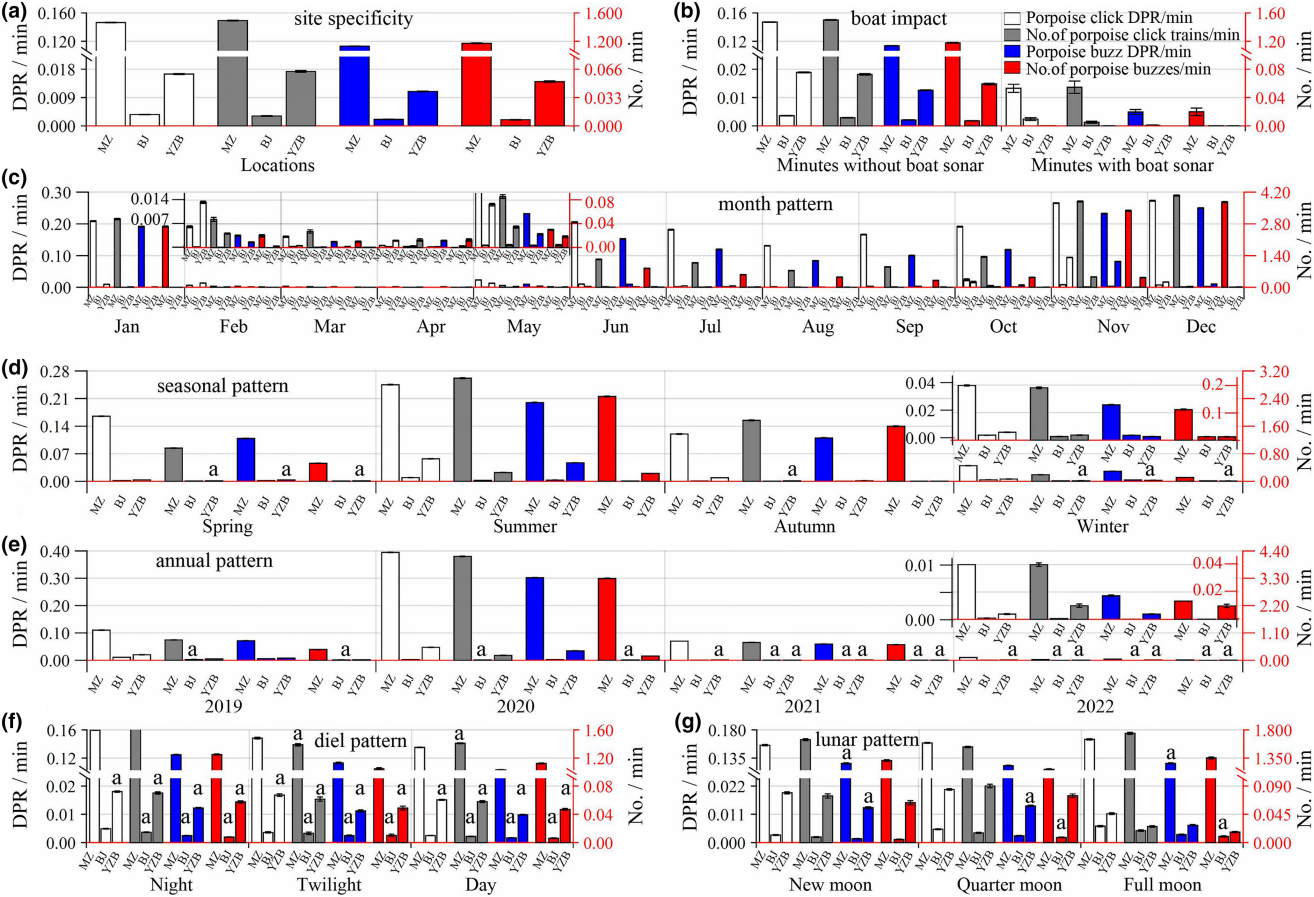
Variation in porpoise biosonar parameters as a function of site fidelity relative to the pooled data (a) and as a function of boat traffic (b), month (c), season (d), year (e), diel (f), and lunar (g) over the data in each site by a generalized linear model. Results are expressed as mean ± standard error of the mean. A subset of data was analyzed for lunar patterns at night. Inset figures depicted a magnified part of the data.

In all biosonar parameters of porpoise click DPRM, number of porpoises click trains per minute, buzzes DPRM, and number of buzzes per minute, a significant spatial pattern was identified, according to the GLM results (Table 2, Table S1, and Figure 4). In particular, all biosonar values at the MZ location are much greater than those at the YZB and BJ sites (post hoc Tukey's HSD Tamhane's T2 post hoc multiple comparison tests; *p* < .001) (Table 2, Table S1 and Figure 4a).

**TABLE 2:** Seven‐way ANOVA of the effects of parameters (site*boat traffic*water flux*diel*month*season*year) on the porpoise click detection positive rate per minute (DPRM) and porpoise buzz DPRM.

Source	Dependent variable	Type III sum of squares	Df	Mean square	*F*	Sig.
Corrected Model	Click DPRM	64,023.94^a^	212	302.00	7614.13	.000
	Buzz DPRM	51,039.19^b^	212	240.75	7974.29	.000
Intercept	Click DPRM	8.42	1	8.42	212.17	<.001
	Buzz DPRM	2.30	1	2.30	76.09	<.001
Site	Click DPRM	9.34	2	4.67	117.69	<.001
	Buzz DPRM	6.41	2	3.20	106.08	<.001
Boat	Click DPRM	1.08	1	1.08	27.21	<.001
	Buzz DPRM	0.57	1	0.57	19.04	<.001
Water flux	Click DPRM	53.67	1	53.67	1353.25	<.001
	Buzz DPRM	6.45	1	6.45	213.60	<.001
Diel	Click DPRM	0.49	2	0.24	6.16	.002
	Buzz DPRM	0.25	2	0.12	4.08	.017
Month	Click DPRM	3.07	11	0.28	7.05	<.001
	Buzz DPRM	2.17	11	0.20	6.53	<.001
Season	Click DPRM	16.13	3	5.38	135.56	<.001
	Buzz DPRM	11.04	3	3.68	121.91	<.001
Year	Click DPRM	4.41	3	1.47	37.10	<.001
	Buzz DPRM	2.06	3	0.69	22.79	<.001
Site*Boat	Click DPRM	6.51	2	3.26	82.09	<.001
	Buzz DPRM	4.33	2	2.16	71.67	<.001
Site*Month	Click DPRM	7.64	20	0.38	9.63	<.001
	Buzz DPRM	6.93	20	0.35	11.48	<.001
Site*Season	Click DPRM	1.95	6	0.32	8.18	<.001
	Buzz DPRM	1.41	6	0.24	7.80	<.001
Site*Year	Click DPRM	3.61	6	0.60	15.17	<.001
	Buzz DPRM	3.48	6	0.58	19.22	<.001
Boat*Month	Click DPRM	3.78	11	0.34	8.66	<.001
	Buzz DPRM	4.03	11	0.37	12.12	<.001
Boat*Year	Click DPRM	2.78	3	0.93	23.40	<.001
	Buzz DPRM	1.20	3	0.40	13.21	<.001
Month*Year	Click DPRM	29.77	18	1.65	41.70	<.001
	Buzz DPRM	34.59	18	1.92	63.65	<.001
Site*Boat*Diel	Click DPRM	29.82	10	2.98	75.17	<.001
	Buzz DPRM	13.15	10	1.31	43.54	<.001
Site*Boat*Month	Click DPRM	9.74	20	0.49	12.28	<.001
	Buzz DPRM	9.64	20	0.48	15.97	<.001
Site*Boat*Season	Click DPRM	5.23	9	0.58	14.65	<.001
	Buzz DPRM	4.46	9	0.50	16.41	<.001
Site*Boat*Year	Click DPRM	11.13	6	1.85	46.76	<.001
	Buzz DPRM	14.99	6	2.50	82.74	<.001
Site*Month*Year	Click DPRM	25.23	18	1.40	35.34	<.001
	Buzz DPRM	20.91	18	1.16	38.47	<.001
Site*Season*Year	Click DPRM	17.72	8	2.21	55.84	<.001
	Buzz DPRM	5.09	8	0.64	21.09	<.001
Boat*Month*Year	Click DPRM	22.08	19	1.16	29.30	<.001
	Buzz DPRM	24.99	19	1.32	43.57	<.001
Month*Season*Year	Click DPRM	66.95	1	66.95	1687.95	.000
	Buzz DPRM	71.63	1	71.63	2372.52	.000
Site*Boat*Month*Year	Click DPRM	33.72	21	1.61	40.48	<.001
	Buzz DPRM	34.86	21	1.66	54.98	<.001
Error	Click DPRM	121,074.90	3,052,587	0.04		
	Buzz DPRM	92,160.29	3,052,587	0.03		
Total	Click DPRM	197,932.00	3,052,800			
	Buzz DPRM	150,632.00	3,052,800			
Corrected Total	Click DPRM	185,098.84	3,052,799			
	Buzz DPRM	143,199.48	3,052,799			

^a^ *R* ^2^ = .35 (Adjusted *R* ^2^ = .35).

^b^ *R* ^2^ = .36 (Adjusted *R* ^2^ = .35).

### 3.2 Temporal patterns

**FIGURE 5:**
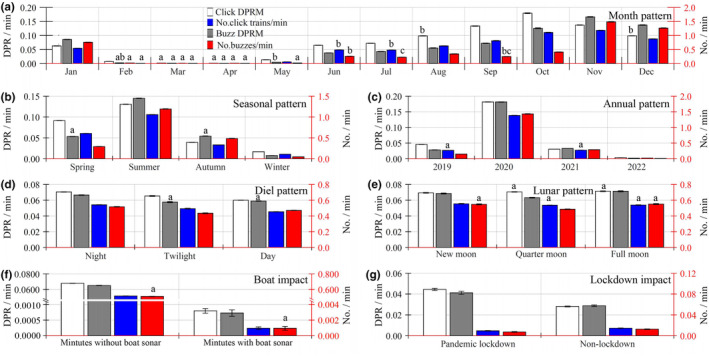
Difference of the porpoise biosonar parameters as a function of the month (a), season (b), year (c), diel (d), lunar (e), boat traffic (f) and pandemic lockdown (g) over the pooled data by a generalized linear model. The results are reported as the mean ± standard error of the mean. Error bars with identical letters represent post hoc multiple comparison tests that failed to detect a statistically significant difference (*p* > .05). A subset of data was analyzed for lunar patterns at night. All figures share the same *y*‐axis label, with porpoise click Detection Positive Rate (DPR) per minute and porpoise buzz DPR per minute sharing the left *y*‐axis label, and the number of porpoise click trains per minute and the number of porpoise buzzes per minute sharing the right *y*‐axis label.

GLM analysis indicates that all biosonar metrics exhibit significant monthly, seasonal, and annual temporal trends (Table 2, Table S1, Figures 4c–e and 5a–c). Significant monthly differences exist for the combined data of the three sites in all four biosonar parameters, with the exception of the porpoise click DPRM between March and April and between August and December (Figure 5a), the number of porpoise click trains per minute between February, March and April and between February and May (Figure 5a), the buzz DPRM between February, March and April and between June and July (Figure 5a), the number of porpoises buzz per minute between February and May and between June and September and between July and December (post hoc Tukey's HSD; *p* > .05) (Table 2, Table S1, Figure 5a). Except for February and April, all four biosonar metrics at the MZ location exhibit statistically significant monthly variations (Table S2, Figure 4c). Significant monthly variation exists in the BJ site for the porpoise click DPRM; however, there is no significant difference in the number of porpoise click trains per minute, buzzes DPRM, and number of buzzes per minute (Table S3, Figure 4c). Significant monthly variation exists for all four biosonar parameters at the YZB location, with the exception of February and May (Table S4, Figure 4c).

All four biosonar characteristics, with the exception of the number of porpoise clicks trains per minute, exhibit substantial seasonal variations between spring and autumn according to the results of a GLM applied to combined data from three sites (post hoc Tukey's HSD; *p* > .05) (Table 2, Table S1 and Figure 5b). All four biosonar metrics at MZ and YZ sites exhibit significant seasonal changes (Tables S2 and S3, Figure 4d). At the YZB location, there are no significant seasonal variations in the number of porpoise click trains per minute between spring, autumn, and winter or in the buzzes DPRM and number of buzzes per minute between spring and winter (Table S4, Figure 4d).

The GLM results for the combined data from the three sites suggest that there are substantial annual differences in all four biosonar metrics, with the exception of buzzes DPRM between 2019 and 2021 (post hoc Tukey's HSD; *p* > .05) (Table 2, Table S1 and Figure 5c). In the MZ site, significant annual differences exist in all four biosonar parameters (Table S2, Figure 4e). In the BJ site, there are large annual differences between the porpoise click DPRM and buzzes DPRM, except between 2019 and 2021 for buzzes DPRM. There were no annual variations in the number of porpoise click trains per minute and buzzes per minute (Table S3, Figure 4e). There are large annual changes in all four biosonar metrics at the YZB site, except between 2021 and 2022 (Table S4, Figure 4e).

### 3.3 Boat traffic

The results of GLM for the pooled data from the three sites indicate that the porpoise clicks DPRM, the number of porpoises click trains per minute and buzzes DPRM during the period with boat traffic were significantly lower than during the period without boat traffic, whereas the number of buzzes per minute did not differ significantly between the periods with and without boat traffic (Table 2, Table S1, and Figure 5f). Specifically, at the MZ location, all biosonar parameters were considerably higher during the period without boat traffic than during the period with boat traffic (Table S2, Figure 4b). In the BJ site, the click DPRM during the period without boat traffic was significantly higher than that in the period with boat traffic. The number of porpoise click trains per minute, buzz DPRM and number of buzzes per minute did not alter significantly between periods with and without boat traffic (Table S3, Figure 4b). In the YZB site, the click DPRM and buzz DPRM during the period without boat traffic was significantly greater than that in the period with boat traffic. Whereas no significant differences were found in the number of porpoise click trains per minute and number of porpoise buzzes per minute between the period with and without boat traffic (Table S4, Figure 4b).

**FIGURE 6:**
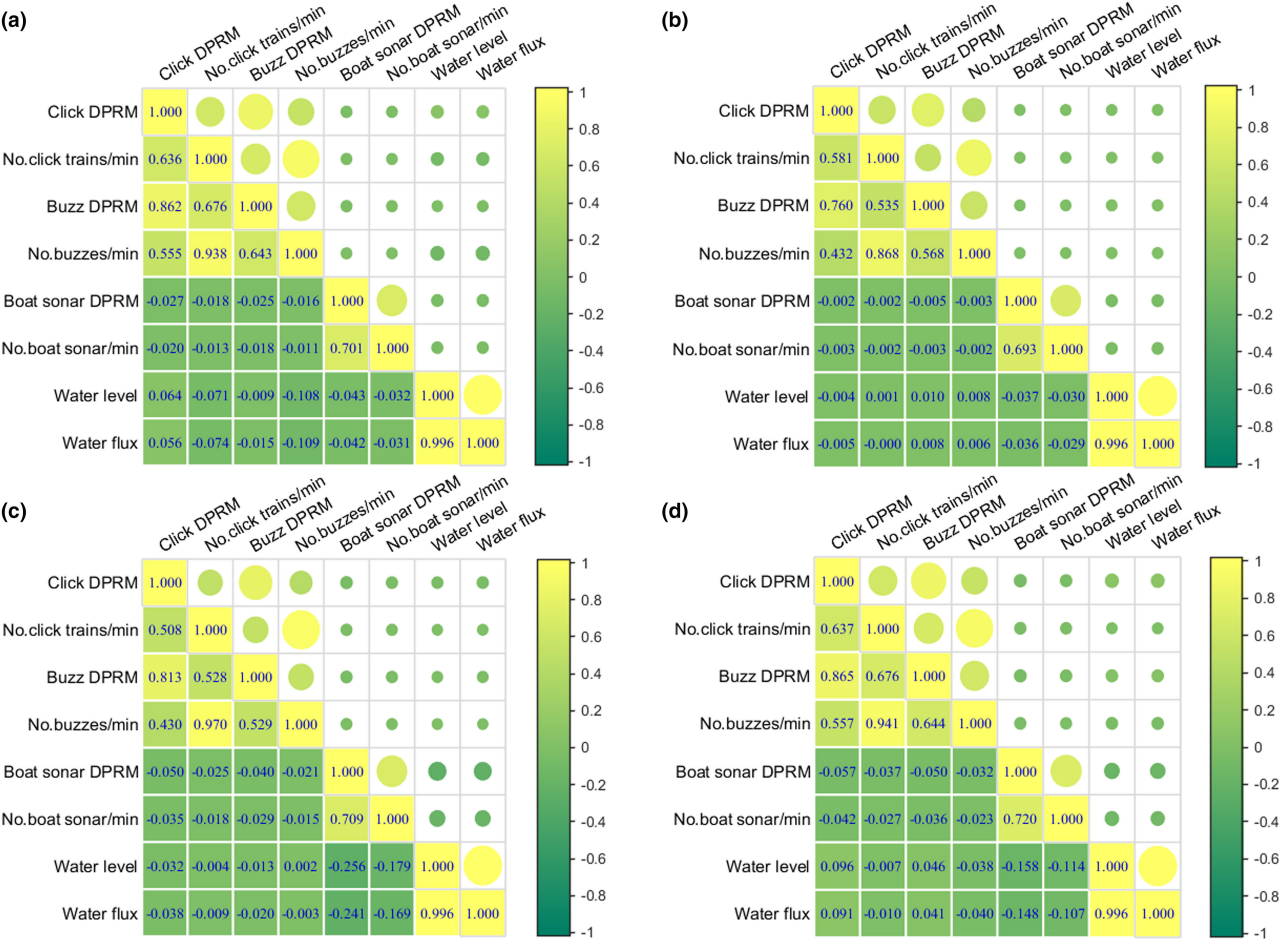
Spearman's rho correlation of porpoise biosonar detection and boat traffic in MZ (a), BJ (b), YZB (c) and over the pooled data (d). *p*‐values were presented for each pairwise linear correlation. DPRM, positive detection rate per minute. The color bar represents the Spearman's rho correlation coefficient derived from correlation analysis. The size of the circle is directly proportional to the correlation coefficient.

There was a significant association between porpoise sonar detections and boat sonar detections at each site and the three sites' combined data (Spearman's rho, *p* < .001, *n* = 1,221,120, 858,240, 973,440 and 3,052,800 for MZ, BJ, YZB and the pooled data, respectively) (Figures 3 and 6). There was a positive correlation between porpoise click train detections and porpoise buzz detections (Spearman's rho, *p* < .001, *n* = 1,221,120, 858,240, 973,440 and 3,052,800 for MZ, BJ, YZB and the pooled data, respectively). In contrast, there was a negative correlation between porpoise click train detections and boat sonar detection (Spearman's rho, *p* < .001, *n* = 1,221,120, 858,240, 973,440 and 3,052,800 for MZ, BJ, YZB and the pooled data, respectively) (Figures 3 and 6). and between porpoise buzz detections and boat sonar detection (Spearman's rho, *p* < .001, *n* = 1,221,120, 858,240, 973,440 and 3,052,800 for MZ, BJ, YZB and the pooled data, respectively) (Figures 3 and 6).

### 3.4 Pandemic lockdown

During the pandemic lockdown, the biosonar activity of porpoises was substantially higher than during the non‐lockdown period. During the pandemic lockdown, all biosonar values were much higher than during the non‐lockdown period (porpoise click DPRM: Mann–Whitney *U*‐test, *z* = 16.73, df = 168,480, *p* < .001; the number of porpoises clicks trains per minute: Mann–Whitney *U*‐test, *z* = 16.68, df = 168,480, *p* < .001; buzzes DPRM: Mann–Whitney *U*‐test, *z* = −5.59, df = 168,480, *p* < .001; the number of buzzes per minute: Mann–Whitney *U*‐test, *z* = −5.59, df = 168,480, *p* < .001) (Figure 5g).

### 3.5 Water flux and water level

The results of GLM for the three locations' combined data reveal that water flux had a substantial effect on all biosonar parameters (Table 2, Table S1). Water flux also had a considerable effect on each site's biosonar characteristics (Tables S2–S4). Since water flux and water level are correlated (Figures 3 and 6), the water level has the same effect on all biosonar parameters as water flux. In the MZ site, water level and water flux were positively and adversely linked with porpoise click and buzz DPRM, respectively. Water level and water flux were significantly negatively connected with porpoise click DPRM and positively correlated with porpoise buzz DPRM at the BJ site. Water level and water flux were significantly negatively linked with porpoise click DPRM and porpoise buzz DPRM at the YZB location. Additionally, water level and water flux were positively connected with porpoise click and buzz DPRM at the YZB location (Figure 6).

### 3.6 Diel and lunar patterns

There was a substantial variation in diel patterns for all biosonar parameters except for the number of porpoise click trains per minute between twilight and daytime, as indicated by the findings of the generalized linear model for the combined data from the three sites (post hoc Tukey's HSD; *p* > .05) (Table 2, Table S1 and Figure 4d). Specifically, nighttime biosonar activity was substantially greater than daytime activity. In addition, significant differences in all biosonar parameters were observed except for the number of porpoises click trains per minute between twilight and day in MZ (post hoc Tukey's HSD; *p* > .05), and for the number of porpoises click trains per minute, buzzes DPRM, and number of buzzes per minute between night and twilight in the BJ site (post hoc Tukey's HSD; *p* > .05) (Figure 4f). There were no significant variations in any biosonar parameter at the YZB site (Table S4).

There was a significant difference in lunar patterns for all biosonar parameters, except for the porpoise click DPRM and the buzzes DPRM between the quarter moon and full moon, and the number of buzzes per minute between the new moon and full moon, according to the results of the GLM for the pooled data from the three sites at night (post hoc Tukey's HSD; *p* > .05) (Table S5 and Figure 5e). Specifically, porpoise click activity was substantially higher during the full moon than at the new moon, whereas porpoise buzz activity was the opposite. Additionally, substantial differences in all biosonar parameters were observed in the MZ site except in the buzzes DPRM between the new moon and full moon (post hoc Tukey's HSD; *p* > .05) (Table S6, Figure 3g), in the BJ site except the number of buzzes per minute between the quarter moon and full moon (post hoc Tukey's HSD; *p* > .05) (Table S7, Figure 4g), in YZB site except the buzz DPRM between the new moon and quarter moon (post hoc Tukey's HSD; *p* > .05) (Table S8, Figure 4g).

### 3.7 Interaction effects

On porpoise biosonar activities, significant interaction effects were detected between boat traffic, hydrological regime of water level and water flux, and light intensity (Table 2 and Tables S1–S8). Significant interaction effects of location, boat traffic, and varied temporal scale are observed in porpoise biosonar detections (Table 2 and Tables S1–S8).

## 4 DISCUSSION

Passive acoustic monitoring is expanding rapidly as a low‐cost and high‐resolution sampling technique for acquiring crucial information on marine animals (Zimmer, 2011). In this study, the proportion of days that are porpoise positive was 64% for the three sites downstream of the Gezhouba Dam, which is less than what was reported between the confluence of Poyang Lake and the Yangtze River (93%) (Duan et al., 2023) and higher than that reported in the Wuhan region of the Yangtze river (43%) (Wang et al., 2024). However, porpoise buzz signals account for 78% of all porpoise click trains over the pooled data of the three study sites, which is significantly greater than that recorded between the confluence of Poyang Lake and the Yangtze River (23%) (Duan et al., 2023) and in the Wuhan region of the Yangtze river (55%) (Wang et al., 2024). The notably increased rate of buzz signals detected in this study, in comparison to other regions along the Yangtze River, warrants further thorough investigation. One potential explanation is that buzz signals may act as indicators of cetacean attempted feeding behavior. However, the actual success of feeding endeavors cannot be guaranteed. Additionally, the Gezhouba Dam serves as the sole corridor for boat entry upstream to the Three Gorges, and the heavy traffic in this area may hinder the successful feeding of the local porpoises, potentially leading to increased feeding attempts. In this investigation, significant site fidelity was observed. Sonar detection of porpoises was much higher in MZ than at other sites. The MZ site was situated at the junction of two rivers beneath the Gezhouba Dam. Yangtze finless porpoises were frequently spotted aggregating at confluence areas where prey biomass and availability were greatest (Duan et al., 2023; Zhang et al., 1993). The finless porpoises in the Yangtze is an opportunistic predator (Kimura et al., 2012). The availability of prey was the key factor influencing the habitat preference of porpoises (Wang, Akamatsu, et al., 2015; Wang et al., 2014). Finless porpoises were discovered more commonly at locations with substantial food resources (Kimura et al., 2012). In this study, the higher porpoise sonar detection in the confluence area, particularly at the MZ site, compared to other nonconfluence areas may be a result of the higher potential prey availability.

**FIGURE 7:**
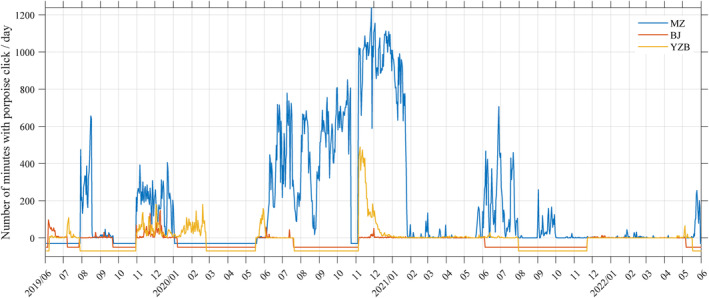
Spatio‐temporal pattern of the number of minutes with porpoise clicks each day for MZ, BJ and YZB sites. Negative values indicate days without a full 24 h recording that were not analyzed in the study. Period with a negative count of porpoise click detections indicate times when the equipment was not deployed. To improve the visual presentation and prevent line overlap, periods without data at the three sites are allocated unique negative values.

No discernible temporal variation in the detection of porpoise sonar was recorded among the three sites during the period of synchronous monitoring (Figure 7). The daily horizontal travel distances of Yangtze finless porpoises exceeded 90 km (Akamatsu et al., 2002). The highest distance between the MZ, BJ, and YZB sites was less than 10 km; therefore theoretically, animal migration across sites is possible. The lack of discernible temporal variation in porpoise sonar detection among the three investigation locations throughout the period of synchronous monitoring suggests that they may each have resident porpoise populations and deserve further research to verification.

### 4.1 Pandemic lockdown and boat traffic

Animals are capable of altering their behavior in reaction to abrupt shifts in human mobility (Bates et al., 2021; Tucker et al., 2023). In order to stop the spread of COVID‐19 at the start of the year 2020, lockdowns were implemented globally, drastically restricting human mobility. The navigation of vessels, along with its potential adverse impacts on porpoises, such as noise interference generated by boats, was also limited. Lockdowns swiftly impacted several spatial habits of wildlife on a global scale (Bates et al., 2021; Tucker et al., 2023). Lockdown also increases biosonar detection of the finless porpoise in the Poyang Lake and Yangtze River confluence region and the Wuhan region of the Yangtze River (Duan et al., 2023; Wang et al., 2024). The same phenomena were found during the lockdown biosonar detection of the finless porpoise in this study.

Threats to the Yangtze finless porpoise include a lack of prey, habitat loss, and pollution (Wang, 2009). The 10‐Year Fishing Ban on the Entire Yangtze River Commencing in 2021 will dramatically relieve the prey shortage (Mei et al., 2020). The green development of the Yangtze River economic belt project can assist in restoring the porpoise's deteriorated habitat (Wang, Duan, Akamatsu, et al., 2021). However, the threat posed by boat traffic shows no signs of abating. In addition to the risk of collision, noise pollution from boat traffic also poses a significant hazard to the porpoise (Wang et al., 2020; Wang, Duan, Akamatsu, et al., 2021), since the busy navigation conditions on the Yangtze River do not appear to improve in the near future. It is necessary to regulate and control boat traffic. Significant changes in dolphin habitat, such as in the western portion of Panama's Dolphin Bay, might result from a shift in boat traffic activities (Gagne et al., 2022). The echolocation signals of Yangtze finless porpoise are distinguished by their narrow‐band characteristics (with a 3 dB bandwidth of approximately 22 kHz) and high frequencies (with a peak frequency around 129 kHz). In contrast, boat noise typically falls within the lower frequency range (<10 kHz). Consequently, there was minimal interference of porpoise sonar by boat sonar, and the absence of buzzes and clicks during boat activity cannot be attributed to a diminished ability to detect them amidst the background noise.

### 4.2 Water level and flux

The hydropower cascade construction, particularly the Three Gorges Dam, has had a substantial impact on the Yangtze River's flow pattern (Li, Liao, et al., 2007; Zhu, 2012). Both water level and water flux had a substantial effect on the biosonar activity of finless porpoise in this investigation. The inherent mechanism may be that hydrological parameters are key determinants of fish species diversity and production (Yang et al., 2020), thereby influencing the food availability for finless porpoise, whereas prey availability is the primary factor that attracts and influences porpoise habitat preference (Wang, Akamatsu, et al., 2015; Wang et al., 2014).

### 4.3 Diel, lunar and temporal patterns

Numerous environmental cycles, such as the diel cycle, lunar phases, and seasons, frequently control the behavior of aquatic animals (Tessmar‐Raible et al., 2011). Long‐term monitoring permits the study of diurnal, monthly, seasonal, and annual dynamics, and long‐term PAM surveys and studies of temporal trends in cetacean biosonar emissions have substantially improved our understanding of their distributions and behavior (Zimmer, 2011). Cetacean biosonar activity varies with light intensity globally and tends to be more acoustically active at night (Caruso et al., 2017; Wang, Nachtigall, et al., 2015). In this investigation, the significantly higher biosonar activity at night than during the day was consistent with that observed in the Pengze area of the Yangtze River (Wang et al., 2014), in the confluence area between Poyang Lake and the Yangtze River (Duan et al., 2023) and in the Wuhan section of the Yangtze River (Wang et al., 2024). The circadian rhythm of biosonar activity in finless porpoises observed in this study did not exhibit as pronounced a pattern compared to that documented in the confluence area between Poyang Lake and the Yangtze River (Duan et al., 2023). However, it closely resembles the pattern observed in the Wuhan section of the Yangtze River (Wang et al., 2024). One potential explanation is that, unlike the well‐established porpoise population in the confluence area between Poyang Lake and the Yangtze River, both the porpoise populations below the Gezhouba Dam and those inhabiting the waters of Wuhan in the Yangtze River are relatively newly formed and are still in the process of adapting to the local environment. The cause of the increase in porpoise sonar detections in late 2020, followed by a rapid decrease in early 2021, is unknown and requires additional investigation.

### 4.4 Water velocity and dams impede porpoise movement upstream of the Gezhouba dam

**FIGURE 8:**
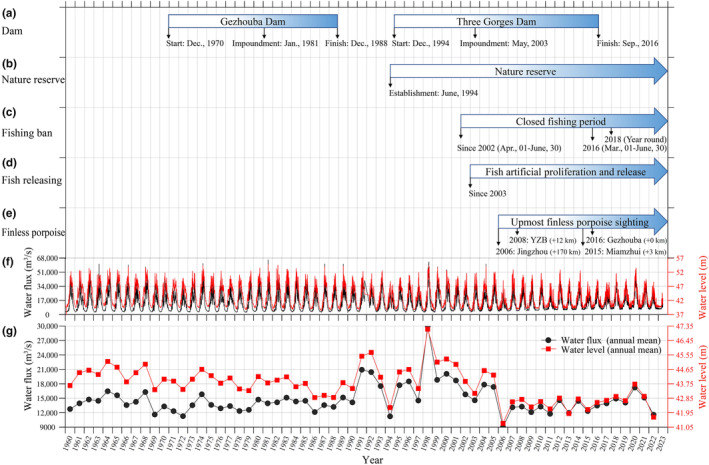
Chronicle of potential key impact factors or events of the dam construction (a), nature reserve construction (b), fish release (c), fishing ban (d) and water flux and water level (f, g) to the distribution of the Yangtze finless porpoise (e) in the downstream of the Gezhouba Dam. Positive values in parentheses indicate the position of the most recent finless porpoise sighting downstream of the Gezhouba Dam, with the Gezhouba Dam serving as the zero‐point reference. The hourly water flux and water level were extracted from the water resources database (https://slt.hubei.gov.cn/sjfb/) using a custom Python program and presented in (f), and the annual averaged result was presented in (g).

The widespread disruption of the longitudinal connection of rivers by dams may limit the migration of cetaceans. Such a dam on the Orinoco River in Venezuela poses a threat to dolphins boto (*Inia geoffrensis*) (Aya et al., 2010). Dams in the Madeira River in Brazil have isolated local boto populations (*I. boliviensis*) (Gravena et al., 2014). Dams in the Araguaia River in Brazil isolated local *I. geoffrensis* populations (Araújo & Silva, 2014). In India, dams on the Ganges River isolated the Susu (*Platanista gangetica gangetica*), whereas, in Pakistan, dams on the Indus River isolated Bhutan (*P. g. minor*) (Smith & Braulik, 2018). Dams on the Mekong River endanger the critically endangered freshwater Mekong dolphin population (*Orcaella brevirostris*) (Brownell et al., 2017). Although there is no available data regarding the uppermost distribution region of the Yangtze finless porpoise within the Yangtze River, historical sightings of the uppermost distribution area of its sympatric species, the river dolphin, the baiji (*Lipotes vexillifer*), occurred approximately 30 km upstream of the Gezhouba Dam near Huangling Temple in 1940, predating the construction of the Gezhouba Dam (Zhou et al., 1977). Additionally, prior to the building of the hydroelectric cascades of the Gezhouba Dam and the Three Gorges Dam, water velocity in the upper Yangtze River, particularly in the Three Gorges region, was exceedingly high. The greatest water velocity in the Three Gorges region in 2002 remained between 3.0 and 4.0 m/s (Chen et al., 2005) following the construction of the Gezhouba Dam, which began in December 1970 and was completed in December 1988 (Figure 8a). Given that the maximum swim speed of Yangtze finless porpoise during diving was 3.74 m/s and the average speed was 1.45 m/s (Akamatsu et al., 2002), finless porpoise may not choose the upstream of the Yangtze River as a habitat. After the construction of the Three Gorges Dam, which was started on December 1994, impoundment on May 2003 and finished on September 2016 (Figure 8a), the Yangtze River flow regime, particularly downstream of Gezhouba Dam, has been dramatically modified (Figure 8f,g) and may facilitate the finless porpoise's habitat in the region.

### 4.5 Prey availability below Gezhouba dam

Prey availability was the primary factor that attracted and influenced the habitat preference of finless porpoise (Wang, Akamatsu, et al., 2015; Wang et al., 2014). Changing hydrological regimes can have significant effects on river ecosystems. Due to the fact that the life cycles of aquatic organisms have adapted effectively to the natural periodic fluctuations of stream hydrological processes, the natural flow regimes are ecologically significant for the reproduction of many fish species, e.g. the four major Chinese carp species, namely black carp (*Mylopharyngodon piceus*), grass carp (*Ctenopharyngodon idellus*), silver carp (*Hypophthalmichthys molitrix*) and bighead carp (*Aristichys nobilis*). Due to changed heat and flow regimes resulting from the construction of the Gezhouba Dam and The Three Gorges Dam, reproduction of these carp has severely reduced, with larval abundance in 2003 being only 24.65% of the value anticipated by the model (Li et al., 2016). In addition, China's Yangtze River biological integrity index has reached the “no fish” level due to overfishing (Ministry‐of‐Agriculture‐and‐Rural‐Affairs, 2021). In order to alleviate the reduction in fisheries resources, compensatory measures were implemented, such as the development of nature reserves and the artificial breeding, stocking, and release of fish. Since July 1994, Yichang has been home to a natural reserve that stretches from the Gezhouba dam to approximately 60 km downstream on the Yangtze River (Figure 8b). In addition, the artificial breeding, stocking, and release of commercially significant fish, such as the four major Chinese carp species, began in the Yangtze River in 2002 (Gui, 2003; Hua, 2002) and was expanded to the Yichang River sections in 2003 (Shi, 2003) (Figure 8e). Since 2002, the area downstream of the Gezhouba Dam has been subject to a fishing ban, with the yearly closed fishing season lasting from 1 April to 30 June. The start date of the closed fishing season was moved to March 1st in 2016 (Mei et al., 2020). As a result, the runoff of fish eggs downstream of the Gezhouba Dam in Yidu city increased by 85.3% between 2017 and 2018 compared to the period between 2009 and 2010, and the runoff of eggs of the four major Chinese carps increased by approximately 13‐fold compared to data from 2005 to 2012 (Chen et al., 2020). In addition, in Yichang, the reserve area fishing restriction has been extended to the entire year since 2018, and a 10‐Year Fishing Ban on the entire Yangtze River began in 2021 (Ministry‐of‐Agriculture‐and‐Rural‐Affairs, 2019). All these activities may enhance prey availability in the Yangtze River downstream of the Gezhouba Dam.

### 4.6 Factors affecting Yichang water porpoise

**FIGURE 9:**
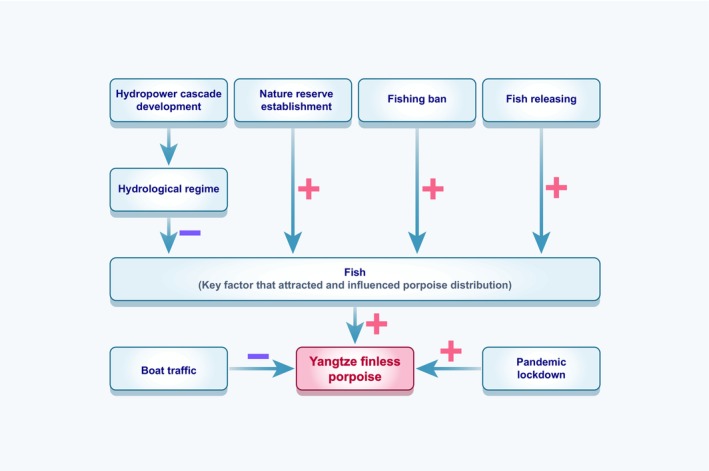
Interaction mechanism of impact factors to the distribution of the Yangtze finless porpoise in the downstream of the Gezhouba Dam.

The uppermost Yangtze finless porpoise populations spread upstream from 170 km downstream of the Gezhouba Dam in 2006, to 12 km downstream of the Gezhouba Dam in 2008, to 3 km downstream of the Gezhouba Dam in 2015, and just below the dam in 2016 are freshly developed. Since 2019 and afterwards in 2022, newborn finless porpoise calves have been spotted in Yichang (He GW, personal communication). The upstream extension of the Yangtze finless porpoise in the downstream of the Gezhouba Dam may be a combined effect of the hydropower cascade development, such as the Gezhouba Dam and the Three Gorges Dam, which altered the hydrological dynamic of the river and affected fish community structure and abundance, as well as the establishment of nature reserves and the artificial breeding, stocking and release and fishing ban that provide and assure prey availability (Figure 9). These factors collectively enhance the activity of the Yangtze finless porpoise in the Yichang water.

### 4.7 Conservation and management implications

Hydrological regimes in the Yangtze River will be further influenced by the planned and ongoing construction of additional dams in the upper reach of the Yangtze River (Wang et al., 2018); therefore, restoration designs that mimic ecologically significant aspects of natural flow regimes should be prioritized to meet the needs of aquatic animals such as porpoise prey.

Cetaceans in freshwater are impacted by noise pollution throughout regions (Wang, Duan, Wang, & Wang, 2021). Noise reduction on the Yangtze River should include establishing no‐navigation zones in the primary habitat of the finless porpoise and restricted navigation zones where assessment and implementation of environmentally suitable navigation speed and vessel traffic flux are required.

Long‐term PAM monitoring at more sites with higher spatial resolution and the development of real‐time monitoring is essential for advancing and facilitating the eventual elucidation of the distribution, behavior patterns, and habitat preference of the porpoise downstream of Gezhouba Dam and in other regions.

## 5 CONCLUSION

Dams contribute to flood protection and energy supply but at the expense of the habitats of freshwater species. After the construction of the Three Gorges Dam and the Gezhouba Dam in the middle stream of the Yangtze River in Yichang, it was noticed that the distribution of the uppermost Yangtze finless porpoise population spread 170 km upstream to below the Gezhouba Dam. Nonetheless, the effects of cascade hydropower developments on the finless porpoise have not been thoroughly described. We evaluated the activity of finless porpoise at three sites downstream of the Gezhouba Dam over several temporal scales by detecting their echolocation clicks using 4 years of data from long‐term passive acoustic monitoring. Over a total of 2120 days of effective surveillance, the daily detection rate of porpoise using biosonar was 64% based on pooled data. We detected a significant temporal and spatial pattern in the biosonar activity of porpoise. The building of hydroelectric cascades, the establishment of nature reserves, the release of fish, and the prohibition on fishing in Yichang may have established the uppermost Yangtze finless porpoise populations. Effective river restoration necessitates a mechanistic understanding of how flow regimes influence local biota and the ecosystem, as well as the variables that jeopardize local endangered species. Our evaluation can assist in establishing conservation priorities for the local porpoise.

## AUTHOR CONTRIBUTIONS

**Zhi‐Tao Wang:** Conceptualization (equal); data curation (equal); formal analysis (equal); funding acquisition (equal); investigation (equal); methodology (equal); project administration (equal); resources (equal); software (equal); supervision (equal); validation (equal); visualization (equal); writing – original draft (equal); writing – review and editing (equal). **Peng‐Xiang Duan:** Data curation (equal); formal analysis (equal); writing – review and editing (equal). **Tomonari Akamatsu:** Methodology (equal); validation (equal); writing – review and editing (equal). **Ke‐Xiong Wang:** Conceptualization (equal); funding acquisition (equal); validation (equal); writing – review and editing (equal). **Ding Wang:** Conceptualization (equal); funding acquisition (equal); validation (equal); writing – review and editing (equal).

## FUNDING INFORMATION

This work was supported by the Ningbo University's Talent Introduction Research Startup Funding (Grant No. ZX2023000154) and the Science and Technology Service Network Initiative of the Chinese Academy of Sciences (KFJ‐STS‐QYZD‐2021‐27‐001).

## CONFLICT OF INTEREST STATEMENT

The authors declare that they have no known competing financial interests or personal relationships that could have appeared to influence the work reported in this paper.

## Supporting information


Tables S1–S8.

## Data Availability

The Data that support the findings of this study are openly available in Dryad at: https://datadryad.org/stash/share/AlJQ27TyNq2wpEfVIUp4n6CthtykqjmVmANjWEc8_pA.
