# Supplementary material for: Temporal and spatial biosonar activity of the recently established uppermost Yangtze finless porpoise population downstream of the Gezhouba Dam: Correlation with hydropower cascade development, shipping, hydrological regime, and light intensity
Source: Ecol Evol. 2024 May 6;14(5):e11346. doi: 10.1002/ece3.11346 (PMC11074705; doi:10.1002/ece3.11346)
Supplement: Supplementary file 1 — Tables S1–S8. [file ECE3-14-e11346-s001.doc]

**Table S1.** Seven-way ANOVA of the effects of parameters (site * boat traffic * water flux * diel * month * season * year) on the number of porpoise click trains per minute and number of buzzes per minute.

| Source | Dependent Variable | Type III Sum of Squares | df | Mean Square | F | Sig. |
| --- | --- | --- | --- | --- | --- | --- |
| Corrected Model | No. click trains/min | 12122443.38^a^ | 202 | 60012.10 | 6167.99 | 0.000 |
|  | No. buzzes/min | 10852429.52^b^ | 202 | 53724.90 | 7194.85 | 0.000 |
| Intercept | No. click trains/min | 889.21 | 1 | 889.21 | 91.39 | <0.001 |
|  | No. buzzes/min | 147.50 | 1 | 147.50 | 19.75 | <0.001 |
| Site | No. click trains/min | 1251.10 | 2 | 625.55 | 64.29 | <0.001 |
|  | No. buzzes/min | 828.53 | 2 | 414.27 | 55.48 | <0.001 |
| Boat | No. click trains/min | 82.18 | 1 | 82.18 | 8.45 | 0.004 |
|  | No. buzzes/min | 27.33 | 1 | 27.33 | 3.66 | 0.056 |
| Water flux | No. click trains/min | 12778.06 | 1 | 12778.06 | 1313.32 | <0.001 |
|  | No. buzzes/min | 595.83 | 1 | 595.83 | 79.79 | <0.001 |
| Diel | No. click trains/min | 859.90 | 2 | 429.95 | 44.19 | <0.001 |
|  | No. buzzes/min | 2645.17 | 2 | 1322.58 | 177.12 | <0.001 |
| Month | No. click trains/min | 550.85 | 11 | 50.08 | 5.15 | <0.001 |
|  | No. buzzes/min | 617.47 | 11 | 56.13 | 7.52 | <0.001 |
| Season | No. click trains/min | 1399.28 | 3 | 466.43 | 47.94 | <0.001 |
|  | No. buzzes/min | 888.16 | 3 | 296.05 | 39.65 | <0.001 |
| Year | No. click trains/min | 450.45 | 3 | 150.15 | 15.43 | <0.001 |
|  | No. buzzes/min | 266.23 | 3 | 88.74 | 11.88 | <0.001 |
| Site * Boat | No. click trains/min | 1044.94 | 2 | 522.47 | 53.70 | <0.001 |
|  | No. buzzes/min | 733.80 | 2 | 366.90 | 49.14 | <0.001 |
| Site * Month | No. click trains/min | 1498.08 | 20 | 74.90 | 7.70 | <0.001 |
|  | No. buzzes/min | 1515.18 | 20 | 75.76 | 10.15 | <0.001 |
| Site * Season | No. click trains/min | 576.35 | 6 | 96.06 | 9.87 | <0.001 |
|  | No. buzzes/min | 572.34 | 6 | 95.39 | 12.77 | <0.001 |
| Site * Year | No. click trains/min | 1590.16 | 6 | 265.03 | 27.24 | <0.001 |
|  | No. buzzes/min | 1704.09 | 6 | 284.02 | 38.04 | <0.001 |
| Boat * Month | No. click trains/min | 898.55 | 11 | 81.69 | 8.40 | <0.001 |
|  | No. buzzes/min | 929.19 | 11 | 84.47 | 11.31 | <0.001 |
| Boat * Year | No. click trains/min | 253.10 | 3 | 84.37 | 8.67 | <0.001 |
|  | No. buzzes/min | 127.48 | 3 | 42.49 | 5.69 | <0.001 |
| Month * Year | No. click trains/min | 7982.86 | 18 | 443.49 | 45.58 | <0.001 |
|  | No. buzzes/min | 8958.83 | 18 | 497.71 | 66.65 | <0.001 |
| Site * Boat * Month | No. click trains/min | 2773.05 | 20 | 138.65 | 14.25 | <0.001 |
|  | No. buzzes/min | 2878.08 | 20 | 143.90 | 19.27 | <0.001 |
| Site * Boat * Season | No. click trains/min | 514.14 | 9 | 57.13 | 5.87 | <0.001 |
|  | No. buzzes/min | 429.83 | 9 | 47.76 | 6.40 | <0.001 |
| Site * Boat * Year | No. click trains/min | 4667.10 | 6 | 777.85 | 79.95 | <0.001 |
|  | No. buzzes/min | 5421.07 | 6 | 903.51 | 121.00 | <0.001 |
| Site * Month * Year | No. click trains/min | 6294.38 | 18 | 349.69 | 35.94 | <0.001 |
|  | No. buzzes/min | 5283.33 | 18 | 293.52 | 39.31 | <0.001 |
| Site * Season * Year | No. click trains/min | 18793.25 | 8 | 2349.16 | 241.44 | 0.000 |
|  | No. buzzes/min | 23132.38 | 8 | 2891.55 | 387.24 | 0.000 |
| Boat * Month * Year | No. click trains/min | 5657.90 | 19 | 297.78 | 30.61 | <0.001 |
|  | No. buzzes/min | 6000.90 | 19 | 315.84 | 42.30 | <0.001 |
| Month * Season * Year | No. click trains/min | 7929.26 | 1 | 7929.26 | 814.96 | <0.001 |
|  | No. buzzes/min | 7621.02 | 1 | 7621.02 | 1020.61 | <0.001 |
| Site * Boat * Month * Year | No. click trains/min | 9098.59 | 21 | 433.27 | 44.53 | <0.001 |
|  | No. buzzes/min | 8545.52 | 21 | 406.93 | 54.50 | <0.001 |
| Error | No. click trains/min | 29700569.51 | 3052597 | 9.73 |  |  |
|  | No. buzzes/min | 22794153.54 | 3052597 | 7.47 |  |  |
| Total | No. click trains/min | 42999173.00 | 3052800 |  |  |  |
|  | No. buzzes/min | 34371254.00 | 3052800 |  |  |  |
| Corrected Total | No. click trains/min | 41823012.89 | 3052799 |  |  |  |
|  | No. buzzes/min | 33646583.05 | 3052799 |  |  |  |

1. R Squared = .29 (Adjusted R Squared = .29)
2. R Squared = .32 (Adjusted R Squared = .32)

**Table S2.** Six-way ANOVA of the effects of parameters (boat traffic * water flux * diel * month * season * year) on the porpoise click detection positive rate per minute (DPRM), number of porpoises click trains per minute, porpoise buzz DPRM and number of porpoises buzz per minute in MZ site.

| Source | Dependent Variable | Type III Sum of Squares | df | Mean Square | F | Sig. |
| --- | --- | --- | --- | --- | --- | --- |
| Corrected Model | Click DPRM | 49798.74a | 157 | 317.19 | 3766.10 | 0.000 |
|  | No. click trains/min | 10914136.20b | 157 | 69516.79 | 2993.19 | 0.000 |
|  | Buzz DPRM | 42057.61c | 157 | 267.88 | 4063.47 | 0.000 |
|  | No. buzzes/min | 10160307.04d | 157 | 64715.33 | 3653.22 | 0.000 |
| Intercept | Click DPRM | 104.79 | 1 | 104.79 | 1244.20 | 0.000 |
|  | No. click trains/min | 13482.24 | 1 | 13482.24 | 580.51 | 0.000 |
|  | Buzz DPRM | 40.86 | 1 | 40.86 | 619.85 | 0.000 |
|  | No. buzzes/min | 3853.01 | 1 | 3853.01 | 217.50 | 0.000 |
| Boat DPRM | Click DPRM | 17.55 | 1 | 17.55 | 208.40 | 0.000 |
|  | No. click trains/min | 2017.80 | 1 | 2017.80 | 86.88 | 0.000 |
|  | Buzz DPRM | 10.88 | 1 | 10.88 | 165.06 | 0.000 |
|  | No. buzzes/min | 1061.60 | 1 | 1061.60 | 59.93 | 0.000 |
| Water flux | Click DPRM | 118.52 | 1 | 118.52 | 1407.27 | 0.000 |
|  | No. click trains/min | 29553.78 | 1 | 29553.78 | 1272.50 | 0.000 |
|  | Buzz DPRM | 20.42 | 1 | 20.42 | 309.69 | 0.000 |
|  | No. buzzes/min | 2532.62 | 1 | 2532.62 | 142.97 | 0.000 |
| Diel | Click DPRM | 36.10 | 2 | 18.05 | 214.30 | 0.000 |
|  | No. click trains/min | 196.39 | 2 | 98.20 | 4.23 | 0.015 |
|  | Buzz DPRM | 19.29 | 2 | 9.64 | 146.30 | 0.000 |
|  | No. buzzes/min | 1601.01 | 2 | 800.51 | 45.19 | 0.000 |
| Month | Click DPRM | 18.87 | 11 | 1.72 | 20.37 | 0.000 |
|  | No. click trains/min | 2179.78 | 11 | 198.16 | 8.53 | 0.000 |
|  | Buzz DPRM | 15.20 | 11 | 1.38 | 20.96 | 0.000 |
|  | No. buzzes/min | 2670.66 | 11 | 242.79 | 13.71 | 0.000 |
| Season | Click DPRM | 11.83 | 3 | 3.94 | 46.83 | 0.000 |
|  | No. click trains/min | 1301.04 | 3 | 433.68 | 18.67 | 0.000 |
|  | Buzz DPRM | 6.21 | 3 | 2.07 | 31.39 | 0.000 |
|  | No. buzzes/min | 486.38 | 3 | 162.13 | 9.15 | 0.000 |
| Year | Click DPRM | 18.80 | 3 | 6.27 | 74.40 | 0.000 |
|  | No. click trains/min | 2869.37 | 3 | 956.46 | 41.18 | 0.000 |
|  | Buzz DPRM | 9.44 | 3 | 3.15 | 47.76 | 0.000 |
|  | No. buzzes/min | 1945.62 | 3 | 648.54 | 36.61 | 0.000 |
| Boat DPRM * Month | Click DPRM | 11.69 | 11 | 1.06 | 12.61 | 0.000 |
|  | No. click trains/min | 2595.50 | 11 | 235.95 | 10.16 | 0.000 |
|  | Buzz DPRM | 10.69 | 11 | 0.97 | 14.75 | 0.000 |
|  | No. buzzes/min | 2660.40 | 11 | 241.85 | 13.65 | 0.000 |
| Boat DPRM * Season | Click DPRM | 3.06 | 3 | 1.02 | 12.10 | 0.000 |
|  | No. click trains/min | 152.81 | 3 | 50.94 | 2.19 | 0.087 |
|  | Buzz DPRM | 2.35 | 3 | 0.78 | 11.89 | 0.000 |
|  | No. buzzes/min | 108.42 | 3 | 36.14 | 2.04 | 0.106 |
| Boat DPRM * Year | Click DPRM | 13.75 | 3 | 4.58 | 54.43 | 0.000 |
|  | No. click trains/min | 1891.43 | 3 | 630.48 | 27.15 | 0.000 |
|  | Buzz DPRM | 8.19 | 3 | 2.73 | 41.41 | 0.000 |
|  | No. buzzes/min | 1200.32 | 3 | 400.11 | 22.59 | 0.000 |
| Month * Year | Click DPRM | 74.19 | 16 | 4.64 | 55.05 | 0.000 |
|  | No. click trains/min | 16991.27 | 16 | 1061.95 | 45.72 | 0.000 |
|  | Buzz DPRM | 79.61 | 16 | 4.98 | 75.47 | 0.000 |
|  | No. buzzes/min | 18432.91 | 16 | 1152.06 | 65.03 | 0.000 |
| Season * Year | Click DPRM | 34.03 | 5 | 6.81 | 80.80 | 0.000 |
|  | No. click trains/min | 21163.46 | 5 | 4232.69 | 182.25 | 0.000 |
|  | Buzz DPRM | 6.37 | 5 | 1.27 | 19.32 | 0.000 |
|  | No. buzzes/min | 27644.87 | 5 | 5528.97 | 312.11 | 0.000 |
| Boat DPRM * Month * Year | Click DPRM | 49.62 | 16 | 3.10 | 36.82 | 0.000 |
|  | No. click trains/min | 12682.79 | 16 | 792.67 | 34.13 | 0.000 |
|  | Buzz DPRM | 52.70 | 16 | 3.29 | 49.96 | 0.000 |
|  | No. buzzes/min | 12545.47 | 16 | 784.09 | 44.26 | 0.000 |
| Diel * Month * Season | Click DPRM | 81.76 | 3 | 27.25 | 323.59 | 0.000 |
|  | No. click trains/min | 13020.29 | 3 | 4340.10 | 186.87 | 0.000 |
|  | Buzz DPRM | 90.03 | 3 | 30.01 | 455.23 | 0.000 |
|  | No. buzzes/min | 12904.39 | 3 | 4301.46 | 242.82 | 0.000 |
| Diel * Month * Year | Click DPRM | 218.13 | 32 | 6.82 | 80.94 | 0.000 |
|  | No. click trains/min | 102960.74 | 32 | 3217.52 | 138.54 | 0.000 |
|  | Buzz DPRM | 194.25 | 32 | 6.07 | 92.08 | 0.000 |
|  | No. buzzes/min | 71105.86 | 32 | 2222.06 | 125.44 | 0.000 |
| Diel * Season * Year | Click DPRM | 29.13 | 10 | 2.91 | 34.58 | 0.000 |
|  | No. click trains/min | 26634.45 | 10 | 2663.45 | 114.68 | 0.000 |
|  | Buzz DPRM | 25.65 | 10 | 2.56 | 38.91 | 0.000 |
|  | No. buzzes/min | 28159.67 | 10 | 2815.97 | 158.96 | 0.000 |
| Error | Click DPRM | 102832.15 | 1220962 | 0.08 |  |  |
|  | No. click trains/min | 28356821.37 | 1220962 | 23.22 |  |  |
|  | Buzz DPRM | 80491.58 | 1220962 | 0.07 |  |  |
|  | No. buzzes/min | 21628824.53 | 1220962 | 17.71 |  |  |
| Total | Click DPRM | 178816.00 | 1221120 |  |  |  |
|  | No. click trains/min | 41993195.00 | 1221120 |  |  |  |
|  | Buzz DPRM | 138187.00 | 1221120 |  |  |  |
|  | No. buzzes/min | 33466080.00 | 1221120 |  |  |  |
| Corrected Total | Click DPRM | 152630.89 | 1221119 |  |  |  |
|  | No. click trains/min | 39270957.57 | 1221119 |  |  |  |
|  | Buzz DPRM | 122549.19 | 1221119 |  |  |  |
|  | No. buzzes/min | 31789131.56 | 1221119 |  |  |  |
| a. R Squared = .326 (Adjusted R Squared = .326) | | | | | | |
| b. R Squared = .278 (Adjusted R Squared = .278) | | | | | | |
| c. R Squared = .343 (Adjusted R Squared = .343) | | | | | | |
| d. R Squared = .320 (Adjusted R Squared = .320) | | | | | | |

**Table S3.** Six-way ANOVA of the effects of parameters (boat traffic * water flux * diel * month * season * year) on the porpoise click detection positive rate per minute (DPRM), number of porpoises click trains per minute, porpoise buzz DPRM and number of porpoises buzz per minute in BJ site.

| Source | Dependent Variable | Type III Sum of Squares | df | Mean Square | F | Sig. |
| --- | --- | --- | --- | --- | --- | --- |
| Corrected Model | Click DPRM | 52.29a | 107 | 0.49 | 138.42 | 0.000 |
|  | No. click trains/min | 471.44b | 107 | 4.41 | 40.49 | 0.000 |
|  | Buzz DPRM | 22.92c | 107 | 0.21 | 104.29 | 0.000 |
|  | No. buzzes/min | 232.38d | 107 | 2.17 | 28.33 | 0.000 |
| Intercept | Click DPRM | 0.99 | 1 | 0.99 | 281.23 | 0.000 |
|  | No. click trains/min | 6.98 | 1 | 6.98 | 64.11 | 0.000 |
|  | Buzz DPRM | 0.17 | 1 | 0.17 | 82.81 | 0.000 |
|  | No. buzzes/min | 2.78 | 1 | 2.78 | 36.32 | 0.000 |
| Boat DPRM | Click DPRM | 0.04 | 1 | 0.04 | 11.39 | 0.001 |
|  | No. click trains/min | 0.01 | 1 | 0.01 | 0.06 | 0.805 |
|  | Buzz DPRM | 0.01 | 1 | 0.01 | 2.65 | 0.103 |
|  | No. buzzes/min | 0.07 | 1 | 0.07 | 0.98 | 0.323 |
| Water flux | Click DPRM | 1.28 | 1 | 1.28 | 362.12 | 0.000 |
|  | No. click trains/min | 16.29 | 1 | 16.29 | 149.71 | 0.000 |
|  | Buzz DPRM | 0.74 | 1 | 0.74 | 358.33 | 0.000 |
|  | No. buzzes/min | 12.29 | 1 | 12.29 | 160.35 | 0.000 |
| Diel | Click DPRM | 0.79 | 2 | 0.39 | 111.58 | 0.000 |
|  | No. click trains/min | 10.02 | 2 | 5.01 | 46.03 | 0.000 |
|  | Buzz DPRM | 0.11 | 2 | 0.05 | 26.51 | 0.000 |
|  | No. buzzes/min | 1.81 | 2 | 0.91 | 11.84 | 0.000 |
| Month | Click DPRM | 0.42 | 11 | 0.04 | 10.75 | 0.000 |
|  | No. click trains/min | 2.12 | 11 | 0.19 | 1.77 | 0.053 |
|  | Buzz DPRM | 0.02 | 11 | 0.00 | 0.84 | 0.598 |
|  | No. buzzes/min | 0.60 | 11 | 0.05 | 0.71 | 0.734 |
| Season | Click DPRM | 2.12 | 3 | 0.71 | 199.85 | 0.000 |
|  | No. click trains/min | 25.73 | 3 | 8.58 | 78.82 | 0.000 |
|  | Buzz DPRM | 1.72 | 3 | 0.57 | 279.88 | 0.000 |
|  | No. buzzes/min | 20.27 | 3 | 6.76 | 88.15 | 0.000 |
| Year | Click DPRM | 0.04 | 3 | 0.01 | 3.34 | 0.018 |
|  | No. click trains/min | 0.31 | 3 | 0.10 | 0.95 | 0.416 |
|  | Buzz DPRM | 0.03 | 3 | 0.01 | 5.02 | 0.002 |
|  | No. buzzes/min | 0.35 | 3 | 0.12 | 1.52 | 0.206 |
| Diel * Month | Click DPRM | 0.26 | 22 | 0.01 | 3.30 | 0.000 |
|  | No. click trains/min | 9.76 | 22 | 0.44 | 4.08 | 0.000 |
|  | Buzz DPRM | 0.08 | 22 | 0.00 | 1.77 | 0.015 |
|  | No. buzzes/min | 6.19 | 22 | 0.28 | 3.67 | 0.000 |
| Diel * Season | Click DPRM | 0.28 | 6 | 0.05 | 13.40 | 0.000 |
|  | No. click trains/min | 3.55 | 6 | 0.59 | 5.44 | 0.000 |
|  | Buzz DPRM | 0.18 | 6 | 0.03 | 14.40 | 0.000 |
|  | No. buzzes/min | 2.93 | 6 | 0.49 | 6.37 | 0.000 |
| Diel * Season * Year | Click DPRM | 12.22 | 22 | 0.56 | 157.37 | 0.000 |
|  | No. click trains/min | 102.26 | 22 | 4.65 | 42.71 | 0.000 |
|  | Buzz DPRM | 4.32 | 22 | 0.20 | 95.58 | 0.000 |
|  | No. buzzes/min | 41.90 | 22 | 1.90 | 24.85 | 0.000 |
| Boat DPRM * Month * Year | Click DPRM | 1.96 | 34 | 0.06 | 16.35 | 0.000 |
|  | No. click trains/min | 38.41 | 34 | 1.13 | 10.38 | 0.000 |
|  | Buzz DPRM | 0.41 | 34 | 0.01 | 5.82 | 0.000 |
|  | No. buzzes/min | 6.30 | 34 | 0.19 | 2.42 | 0.000 |
| Error | Click DPRM | 3029.56 | 858132 | 0.00 |  |  |
|  | No. click trains/min | 93379.94 | 858132 | 0.11 |  |  |
|  | Buzz DPRM | 1762.35 | 858132 | 0.00 |  |  |
|  | No. buzzes/min | 65785.06 | 858132 | 0.08 |  |  |
| Total | Click DPRM | 3093.00 | 858240 |  |  |  |
|  | No. click trains/min | 93966.00 | 858240 |  |  |  |
|  | Buzz DPRM | 1789.00 | 858240 |  |  |  |
|  | No. buzzes/min | 66062.00 | 858240 |  |  |  |
| Corrected Total | Click DPRM | 3081.85 | 858239 |  |  |  |
|  | No. click trains/min | 93851.39 | 858239 |  |  |  |
|  | Buzz DPRM | 1785.27 | 858239 |  |  |  |
|  | No. buzzes/min | 66017.44 | 858239 |  |  |  |
| a. R Squared = .017 (Adjusted R Squared = .017) | | | | | | |
| b. R Squared = .005 (Adjusted R Squared = .005) | | | | | | |
| c. R Squared = .013 (Adjusted R Squared = .013) | | | | | | |
| d. R Squared = .004 (Adjusted R Squared = .003) | | | | | | |

**Table S4.** Six-way ANOVA of the effects of parameters (boat traffic * water flux * diel * month * season * year) on the porpoise click detection positive rate per minute (DPRM), number of porpoises click trains per minute, porpoise buzz DPRM and number of porpoises buzz per minute in YZB site.

| Source | Dependent Variable | Type III Sum of Squares | df | Mean Square | F | Sig. |
| --- | --- | --- | --- | --- | --- | --- |
| Corrected Model | Click DPRM | 1394.75a | 37 | 37.70 | 2554.43 | 0.000 |
|  | No. click trains/min | 40739.69b | 37 | 1101.07 | 1235.69 | 0.000 |
|  | Buzz DPRM | 1302.44c | 37 | 35.20 | 3709.54 | 0.000 |
|  | No. buzzes/min | 39702.75d | 37 | 1073.05 | 1310.84 | 0.000 |
| Intercept | Click DPRM | 0.65 | 1 | 0.65 | 43.90 | 0.000 |
|  | No. click trains/min | 137.18 | 1 | 137.18 | 153.96 | 0.000 |
|  | Buzz DPRM | 0.04 | 1 | 0.04 | 3.76 | 0.053 |
|  | No. buzzes/min | 183.80 | 1 | 183.80 | 224.54 | 0.000 |
| Boat DPRM | Click DPRM | 0.13 | 1 | 0.13 | 9.08 | 0.003 |
|  | No. click trains/min | 1.38 | 1 | 1.38 | 1.55 | 0.213 |
|  | Buzz DPRM | 0.04 | 1 | 0.04 | 3.89 | 0.049 |
|  | No. buzzes/min | 0.67 | 1 | 0.67 | 0.82 | 0.364 |
| Water flux | Click DPRM | 4.53 | 1 | 4.53 | 307.16 | 0.000 |
|  | No. click trains/min | 580.72 | 1 | 580.72 | 651.72 | 0.000 |
|  | Buzz DPRM | 4.79 | 1 | 4.79 | 504.85 | 0.000 |
|  | No. buzzes/min | 585.23 | 1 | 585.23 | 714.92 | 0.000 |
| Diel | Click DPRM | 0.05 | 2 | 0.03 | 1.85 | 0.158 |
|  | No. click trains/min | 0.77 | 2 | 0.39 | 0.43 | 0.649 |
|  | Buzz DPRM | 0.02 | 2 | 0.01 | 0.90 | 0.408 |
|  | No. buzzes/min | 0.59 | 2 | 0.29 | 0.36 | 0.699 |
| Month | Click DPRM | 130.54 | 9 | 14.50 | 982.88 | 0.000 |
|  | No. click trains/min | 4001.09 | 9 | 444.57 | 498.92 | 0.000 |
|  | Buzz DPRM | 110.99 | 9 | 12.33 | 1299.56 | 0.000 |
|  | No. buzzes/min | 3912.11 | 9 | 434.68 | 531.01 | 0.000 |
| Season | Click DPRM | 5.76 | 3 | 1.92 | 130.05 | 0.000 |
|  | No. click trains/min | 174.07 | 3 | 58.02 | 65.12 | 0.000 |
|  | Buzz DPRM | 3.66 | 3 | 1.22 | 128.63 | 0.000 |
|  | No. buzzes/min | 144.68 | 3 | 48.23 | 58.91 | 0.000 |
| Year | Click DPRM | 190.22 | 3 | 63.41 | 4296.69 | 0.000 |
|  | No. click trains/min | 3201.60 | 3 | 1067.20 | 1197.68 | 0.000 |
|  | Buzz DPRM | 102.31 | 3 | 34.10 | 3593.85 | 0.000 |
|  | No. buzzes/min | 2484.07 | 3 | 828.02 | 1011.52 | 0.000 |
| Season * Year | Click DPRM | 3.23 | 5 | 0.65 | 43.73 | 0.000 |
|  | No. click trains/min | 55.86 | 5 | 11.17 | 12.54 | 0.000 |
|  | Buzz DPRM | 3.28 | 5 | 0.66 | 69.10 | 0.000 |
|  | No. buzzes/min | 50.32 | 5 | 10.06 | 12.29 | 0.000 |
| Month * Year | Click DPRM | 282.17 | 11 | 25.65 | 1738.28 | 0.000 |
|  | No. click trains/min | 10646.00 | 11 | 967.82 | 1086.15 | 0.000 |
|  | Buzz DPRM | 270.11 | 11 | 24.56 | 2587.70 | 0.000 |
|  | No. buzzes/min | 10076.31 | 11 | 916.03 | 1119.03 | 0.000 |
| Error | Click DPRM | 14364.51 | 973402 | 0.01 |  |  |
|  | No. click trains/min | 867357.50 | 973402 | 0.89 |  |  |
|  | Buzz DPRM | 9236.92 | 973402 | 0.01 |  |  |
|  | No. buzzes/min | 796821.48 | 973402 | 0.82 |  |  |
| Total | Click DPRM | 16023.00 | 973440 |  |  |  |
|  | No. click trains/min | 912012.00 | 973440 |  |  |  |
|  | Buzz DPRM | 10656.00 | 973440 |  |  |  |
|  | No. buzzes/min | 839112.00 | 973440 |  |  |  |
| Corrected Total | Click DPRM | 15759.26 | 973439 |  |  |  |
|  | No. click trains/min | 908097.18 | 973439 |  |  |  |
|  | Buzz DPRM | 10539.35 | 973439 |  |  |  |
|  | No. buzzes/min | 836524.23 | 973439 |  |  |  |
| a. R Squared = .089 (Adjusted R Squared = .088) | | | | | | |
| b. R Squared = .045 (Adjusted R Squared = .045) | | | | | | |
| c. R Squared = .124 (Adjusted R Squared = .124) | | | | | | |
| d. R Squared = .047 (Adjusted R Squared = .047) | | | | | | |

**Table S5.** Seven-way ANOVA of the effects of parameters (site * boat traffic * water flux * lunar * month * season * year) on the porpoise click detection positive rate per minute (DPRM), number of porpoises click trains per minute, porpoise buzz DPRM and number of porpoises buzz per minute for the pooled data of the three sites at night.

| Source | Dependent variable | Type III Sum of Squares | df | Mean Square | F | Sig. |
| --- | --- | --- | --- | --- | --- | --- |
| Corrected Model | Click DPRM | 29725.26 | 188 | 158.11 | 3730.07 | 0.000 |
|  | No. click trains/min | 4793781.29 | 188 | 25498.84 | 2502.86 | 0.000 |
|  | Buzz DPRM | 23569.18 | 188 | 125.37 | 3812.63 | 0.000 |
|  | No. buzzes/min | 4218106.86 | 188 | 22436.74 | 2908.89 | 0.000 |
| Intercept | Click DPRM | 8.60 | 1 | 8.60 | 203.00 | 0.000 |
|  | No. click trains/min | 1066.90 | 1 | 1066.90 | 104.72 | 0.000 |
|  | Buzz DPRM | 1.83 | 1 | 1.83 | 55.55 | 0.000 |
|  | No. buzzes/min | 171.49 | 1 | 171.49 | 22.23 | 0.000 |
| Site | Click DPRM | 7.00 | 2 | 3.50 | 82.51 | 0.000 |
|  | No. click trains/min | 646.44 | 2 | 323.22 | 31.73 | 0.000 |
|  | Buzz DPRM | 4.41 | 2 | 2.20 | 67.04 | 0.000 |
|  | No. buzzes/min | 418.77 | 2 | 209.38 | 27.15 | 0.000 |
| Boat DPRM | Click DPRM | 0.74 | 1 | 0.74 | 17.57 | 0.000 |
|  | No. click trains/min | 76.76 | 1 | 76.76 | 7.53 | 0.006 |
|  | Buzz DPRM | 0.52 | 1 | 0.52 | 15.86 | 0.000 |
|  | No. buzzes/min | 23.81 | 1 | 23.81 | 3.09 | 0.079 |
| Water flux | Click DPRM | 37.38 | 1 | 37.38 | 881.88 | 0.000 |
|  | No. click trains/min | 11766.04 | 1 | 11766.04 | 1154.91 | 0.000 |
|  | Buzz DPRM | 3.80 | 1 | 3.80 | 115.53 | 0.000 |
|  | No. buzzes/min | 1061.46 | 1 | 1061.46 | 137.62 | 0.000 |
| Lunar | Click DPRM | 0.90 | 2 | 0.45 | 10.64 | 0.000 |
|  | No. click trains/min | 1564.24 | 2 | 782.12 | 76.77 | 0.000 |
|  | Buzz DPRM | 3.59 | 2 | 1.80 | 54.59 | 0.000 |
|  | No. buzzes/min | 1840.03 | 2 | 920.02 | 119.28 | 0.000 |
| Month | Click DPRM | 8.81 | 11 | 0.80 | 18.90 | 0.000 |
|  | No. click trains/min | 696.05 | 11 | 63.28 | 6.21 | 0.000 |
|  | Buzz DPRM | 4.86 | 11 | 0.44 | 13.45 | 0.000 |
|  | No. buzzes/min | 383.60 | 11 | 34.87 | 4.52 | 0.000 |
| Season | Click DPRM | 199.11 | 3 | 66.37 | 1565.76 | 0.000 |
|  | No. click trains/min | 25374.47 | 3 | 8458.16 | 830.22 | 0.000 |
|  | Buzz DPRM | 144.17 | 3 | 48.06 | 1461.48 | 0.000 |
|  | No. buzzes/min | 12991.58 | 3 | 4330.53 | 561.45 | 0.000 |
| Year | Click DPRM | 2.54 | 3 | 0.85 | 19.94 | 0.000 |
|  | No. click trains/min | 171.80 | 3 | 57.27 | 5.62 | 0.001 |
|  | Buzz DPRM | 1.06 | 3 | 0.35 | 10.71 | 0.000 |
|  | No. buzzes/min | 75.89 | 3 | 25.30 | 3.28 | 0.020 |
| Site * Boat DPRM | Click DPRM | 1.36 | 2 | 0.68 | 16.02 | 0.000 |
|  | No. click trains/min | 207.99 | 2 | 103.99 | 10.21 | 0.000 |
|  | Buzz DPRM | 0.83 | 2 | 0.41 | 12.56 | 0.000 |
|  | No. buzzes/min | 138.86 | 2 | 69.43 | 9.00 | 0.000 |
| Site * Month | Click DPRM | 15.07 | 20 | 0.75 | 17.77 | 0.000 |
|  | No. click trains/min | 2991.55 | 20 | 149.58 | 14.68 | 0.000 |
|  | Buzz DPRM | 17.05 | 20 | 0.85 | 25.92 | 0.000 |
|  | No. buzzes/min | 3054.70 | 20 | 152.73 | 19.80 | 0.000 |
| Site * Season | Click DPRM | 249.03 | 6 | 41.50 | 979.14 | 0.000 |
|  | No. click trains/min | 23068.69 | 6 | 3844.78 | 377.39 | 0.000 |
|  | Buzz DPRM | 193.15 | 6 | 32.19 | 979.02 | 0.000 |
|  | No. buzzes/min | 13779.29 | 6 | 2296.55 | 297.74 | 0.000 |
| Site * Year | Click DPRM | 1.34 | 6 | 0.22 | 5.27 | 0.000 |
|  | No. click trains/min | 428.32 | 6 | 71.39 | 7.01 | 0.000 |
|  | Buzz DPRM | 1.29 | 6 | 0.22 | 6.55 | 0.000 |
|  | No. buzzes/min | 395.22 | 6 | 65.87 | 8.54 | 0.000 |
| Boat DPRM * Month | Click DPRM | 2.32 | 11 | 0.21 | 4.97 | 0.000 |
|  | No. click trains/min | 449.17 | 11 | 40.83 | 4.01 | 0.000 |
|  | Buzz DPRM | 2.23 | 11 | 0.20 | 6.18 | 0.000 |
|  | No. buzzes/min | 475.26 | 11 | 43.21 | 5.60 | 0.000 |
| Boat DPRM * Year | Click DPRM | 1.17 | 3 | 0.39 | 9.20 | 0.000 |
|  | No. click trains/min | 100.50 | 3 | 33.50 | 3.29 | 0.020 |
|  | Buzz DPRM | 0.49 | 3 | 0.16 | 4.95 | 0.002 |
|  | No. buzzes/min | 34.13 | 3 | 11.38 | 1.47 | 0.219 |
| Month * Year | Click DPRM | 13.44 | 18 | 0.75 | 17.62 | 0.000 |
|  | No. click trains/min | 3173.31 | 18 | 176.30 | 17.30 | 0.000 |
|  | Buzz DPRM | 15.34 | 18 | 0.85 | 25.92 | 0.000 |
|  | No. buzzes/min | 4377.91 | 18 | 243.22 | 31.53 | 0.000 |
| Site * Boat DPRM * Month | Click DPRM | 6.31 | 19 | 0.33 | 7.83 | 0.000 |
|  | No. click trains/min | 1552.13 | 19 | 81.69 | 8.02 | 0.000 |
|  | Buzz DPRM | 6.43 | 19 | 0.34 | 10.29 | 0.000 |
|  | No. buzzes/min | 1601.00 | 19 | 84.26 | 10.92 | 0.000 |
| Site * Boat DPRM * Year | Click DPRM | 5.77 | 6 | 0.96 | 22.68 | 0.000 |
|  | No. click trains/min | 1906.01 | 6 | 317.67 | 31.18 | 0.000 |
|  | Buzz DPRM | 7.96 | 6 | 1.33 | 40.35 | 0.000 |
|  | No. buzzes/min | 2287.91 | 6 | 381.32 | 49.44 | 0.000 |
| Site * Month * Year | Click DPRM | 16.74 | 18 | 0.93 | 21.94 | 0.000 |
|  | No. click trains/min | 3307.37 | 18 | 183.74 | 18.04 | 0.000 |
|  | Buzz DPRM | 9.79 | 18 | 0.54 | 16.54 | 0.000 |
|  | No. buzzes/min | 2699.06 | 18 | 149.95 | 19.44 | 0.000 |
| Site * Season * Year | Click DPRM | 8.01 | 8 | 1.00 | 23.63 | 0.000 |
|  | No. click trains/min | 1617.89 | 8 | 202.24 | 19.85 | 0.000 |
|  | Buzz DPRM | 3.00 | 8 | 0.37 | 11.39 | 0.000 |
|  | No. buzzes/min | 5536.17 | 8 | 692.02 | 89.72 | 0.000 |
| Boat DPRM * Month * Year | Click DPRM | 7.48 | 19 | 0.39 | 9.29 | 0.000 |
|  | No. click trains/min | 1656.34 | 19 | 87.18 | 8.56 | 0.000 |
|  | Buzz DPRM | 8.86 | 19 | 0.47 | 14.18 | 0.000 |
|  | No. buzzes/min | 1977.32 | 19 | 104.07 | 13.49 | 0.000 |
| Month * Season * Year | Click DPRM | 54.42 | 1 | 54.42 | 1283.81 | 0.000 |
|  | No. click trains/min | 10937.89 | 1 | 10937.89 | 1073.62 | 0.000 |
|  | Buzz DPRM | 65.30 | 1 | 65.30 | 1985.99 | 0.000 |
|  | No. buzzes/min | 11446.32 | 1 | 11446.32 | 1484.00 | 0.000 |
| Site * Boat DPRM * Month * Year | Click DPRM | 10.12 | 17 | 0.60 | 14.04 | 0.000 |
|  | No. click trains/min | 2320.61 | 17 | 136.51 | 13.40 | 0.000 |
|  | Buzz DPRM | 10.73 | 17 | 0.63 | 19.20 | 0.000 |
|  | No. buzzes/min | 2291.58 | 17 | 134.80 | 17.48 | 0.000 |
| Error | Click DPRM | 54493.21 | 1285559 | 0.04 |  |  |
|  | No. click trains/min | 13097121.62 | 1285559 | 10.19 |  |  |
|  | Buzz DPRM | 42272.15 | 1285559 | 0.03 |  |  |
|  | No. buzzes/min | 9915732.06 | 1285559 | 7.71 |  |  |
| Total | Click DPRM | 90603.00 | 1285748 |  |  |  |
|  | No. click trains/min | 18460047.00 | 1285748 |  |  |  |
|  | Buzz DPRM | 69610.00 | 1285748 |  |  |  |
|  | No. buzzes/min | 14477702.00 | 1285748 |  |  |  |
| Corrected Total | Click DPRM | 84218.46 | 1285747 |  |  |  |
|  | No. click trains/min | 17890902.90 | 1285747 |  |  |  |
|  | Buzz DPRM | 65841.34 | 1285747 |  |  |  |
|  | No. buzzes/min | 14133838.92 | 1285747 |  |  |  |
| a. R Squared = .353 (Adjusted R Squared = .353) | | | | | | |
| b. R Squared = .268 (Adjusted R Squared = .268) | | | | | | |
| c. R Squared = .358 (Adjusted R Squared = .358) | | | | | | |
| d. R Squared = .298 (Adjusted R Squared = .298) | | | | | | |

**Table S6.** Five-way ANOVA of the effects of parameters (boat traffic * lunar * month * season * year) on the porpoise click detection positive rate per minute (DPRM), number of porpoises click trains per minute, porpoise buzz DPRM and number of porpoises buzz per minute in MZ site at night.

| Source | Dependent Variable | Type III Sum of Squares | df | Mean Square | F | Sig. |
| --- | --- | --- | --- | --- | --- | --- |
| Corrected Model | Click DPRM | 23408.65a | 137 | 170.87 | 1935.23 | 0.000 |
|  | No. click trains/min | 4226075.82b | 137 | 30847.27 | 1267.92 | 0.000 |
|  | Buzz DPRM | 19676.15c | 137 | 143.62 | 2030.62 | 0.000 |
|  | No. buzzes/min | 3929358.05d | 137 | 28681.45 | 1573.58 | 0.000 |
| Intercept | Click DPRM | 25.83 | 1 | 25.83 | 292.54 | 0.000 |
|  | No. click trains/min | 1788.88 | 1 | 1788.88 | 73.53 | 0.000 |
|  | Buzz DPRM | 13.81 | 1 | 13.81 | 195.32 | 0.000 |
|  | No. buzzes/min | 996.01 | 1 | 996.01 | 54.65 | 0.000 |
| Boat DPRM | Click DPRM | 3.55 | 1 | 3.55 | 40.16 | 0.000 |
|  | No. click trains/min | 489.47 | 1 | 489.47 | 20.12 | 0.000 |
|  | Buzz DPRM | 2.72 | 1 | 2.72 | 38.39 | 0.000 |
|  | No. buzzes/min | 241.74 | 1 | 241.74 | 13.26 | 0.000 |
| Lunar | Click DPRM | 5.34 | 2 | 2.67 | 30.26 | 0.000 |
|  | No. click trains/min | 2748.61 | 2 | 1374.31 | 56.49 | 0.000 |
|  | Buzz DPRM | 3.69 | 2 | 1.84 | 26.08 | 0.000 |
|  | No. buzzes/min | 1547.50 | 2 | 773.75 | 42.45 | 0.000 |
| Month | Click DPRM | 18.54 | 11 | 1.69 | 19.09 | 0.000 |
|  | No. click trains/min | 1763.43 | 11 | 160.31 | 6.59 | 0.000 |
|  | Buzz DPRM | 14.53 | 11 | 1.32 | 18.67 | 0.000 |
|  | No. buzzes/min | 2037.78 | 11 | 185.25 | 10.16 | 0.000 |
| Season | Click DPRM | 82.22 | 3 | 27.41 | 310.41 | 0.000 |
|  | No. click trains/min | 6921.43 | 3 | 2307.14 | 94.83 | 0.000 |
|  | Buzz DPRM | 67.47 | 3 | 22.49 | 317.96 | 0.000 |
|  | No. buzzes/min | 4586.05 | 3 | 1528.68 | 83.87 | 0.000 |
| Year | Click DPRM | 4.61 | 3 | 1.54 | 17.42 | 0.000 |
|  | No. click trains/min | 510.40 | 3 | 170.13 | 6.99 | 0.000 |
|  | Buzz DPRM | 3.12 | 3 | 1.04 | 14.69 | 0.000 |
|  | No. buzzes/min | 321.58 | 3 | 107.19 | 5.88 | 0.001 |
| Boat DPRM * Month | Click DPRM | 9.64 | 11 | 0.88 | 9.93 | 0.000 |
|  | No. click trains/min | 2173.47 | 11 | 197.59 | 8.12 | 0.000 |
|  | Buzz DPRM | 8.55 | 11 | 0.78 | 10.99 | 0.000 |
|  | No. buzzes/min | 2073.95 | 11 | 188.54 | 10.34 | 0.000 |
| Boat DPRM * Year | Click DPRM | 3.76 | 3 | 1.25 | 14.19 | 0.000 |
|  | No. click trains/min | 562.35 | 3 | 187.45 | 7.70 | 0.000 |
|  | Buzz DPRM | 2.14 | 3 | 0.71 | 10.08 | 0.000 |
|  | No. buzzes/min | 332.36 | 3 | 110.79 | 6.08 | 0.000 |
| Month * Season | Click DPRM | 26.76 | 1 | 26.76 | 303.14 | 0.000 |
|  | No. click trains/min | 3208.36 | 1 | 3208.36 | 131.87 | 0.000 |
|  | Buzz DPRM | 35.39 | 1 | 35.39 | 500.38 | 0.000 |
|  | No. buzzes/min | 3815.71 | 1 | 3815.71 | 209.35 | 0.000 |
| Month * Year | Click DPRM | 42.33 | 16 | 2.65 | 29.97 | 0.000 |
|  | No. click trains/min | 7871.19 | 16 | 491.95 | 20.22 | 0.000 |
|  | Buzz DPRM | 43.22 | 16 | 2.70 | 38.19 | 0.000 |
|  | No. buzzes/min | 8905.45 | 16 | 556.59 | 30.54 | 0.000 |
| Month * Lunar | Click DPRM | 465.26 | 22 | 21.15 | 239.53 | 0.000 |
|  | No. click trains/min | 76994.98 | 22 | 3499.77 | 143.85 | 0.000 |
|  | Buzz DPRM | 413.85 | 22 | 18.81 | 265.97 | 0.000 |
|  | No. buzzes/min | 65756.92 | 22 | 2988.95 | 163.99 | 0.000 |
| Season * Year | Click DPRM | 24.01 | 5 | 4.80 | 54.40 | 0.000 |
|  | No. click trains/min | 5568.77 | 5 | 1113.75 | 45.78 | 0.000 |
|  | Buzz DPRM | 37.02 | 5 | 7.40 | 104.69 | 0.000 |
|  | No. buzzes/min | 3832.57 | 5 | 766.51 | 42.05 | 0.000 |
| Season * Lunar | Click DPRM | 46.07 | 6 | 7.68 | 86.97 | 0.000 |
|  | No. click trains/min | 8773.85 | 6 | 1462.31 | 60.11 | 0.000 |
|  | Buzz DPRM | 42.99 | 6 | 7.16 | 101.30 | 0.000 |
|  | No. buzzes/min | 2085.53 | 6 | 347.59 | 19.07 | 0.000 |
| Boat DPRM * Month * Year | Click DPRM | 15.98 | 16 | 1.00 | 11.31 | 0.000 |
|  | No. click trains/min | 3924.14 | 16 | 245.26 | 10.08 | 0.000 |
|  | Buzz DPRM | 17.50 | 16 | 1.09 | 15.46 | 0.000 |
|  | No. buzzes/min | 4211.12 | 16 | 263.19 | 14.44 | 0.000 |
| Month * Year * Lunar | Click DPRM | 293.12 | 20 | 14.66 | 165.99 | 0.000 |
|  | No. click trains/min | 58172.72 | 20 | 2908.64 | 119.55 | 0.000 |
|  | Buzz DPRM | 262.47 | 20 | 13.12 | 185.55 | 0.000 |
|  | No. buzzes/min | 55646.41 | 20 | 2782.32 | 152.65 | 0.000 |
| Season * Year * Lunar | Click DPRM | 20.80 | 3 | 6.93 | 78.52 | 0.000 |
|  | No. click trains/min | 7111.47 | 3 | 2370.49 | 97.43 | 0.000 |
|  | Buzz DPRM | 22.88 | 3 | 7.63 | 107.81 | 0.000 |
|  | No. buzzes/min | 2053.65 | 3 | 684.55 | 37.56 | 0.000 |
| Error | Click DPRM | 45012.25 | 509810 | 0.09 |  |  |
|  | No. click trains/min | 12403195.10 | 509810 | 24.33 |  |  |
|  | Buzz DPRM | 36057.78 | 509810 | 0.07 |  |  |
|  | No. buzzes/min | 9292241.62 | 509810 | 18.23 |  |  |
| Total | Click DPRM | 81421.00 | 509948 |  |  |  |
|  | No. click trains/min | 17951979.00 | 509948 |  |  |  |
|  | Buzz DPRM | 63688.00 | 509948 |  |  |  |
|  | No. buzzes/min | 14020968.00 | 509948 |  |  |  |
| Corrected Total | Click DPRM | 68420.89 | 509947 |  |  |  |
|  | No. click trains/min | 16629270.93 | 509947 |  |  |  |
|  | Buzz DPRM | 55733.93 | 509947 |  |  |  |
|  | No. buzzes/min | 13221599.67 | 509947 |  |  |  |
| a. R Squared = .342 (Adjusted R Squared = .342) | | | | | | |
| b. R Squared = .254 (Adjusted R Squared = .254) | | | | | | |
| c. R Squared = .353 (Adjusted R Squared = .353) | | | | | | |
| d. R Squared = .297 (Adjusted R Squared = .297) | | | | | | |

**Table S7.** Six-way ANOVA of the effects of parameters (boat traffic * lunar * water flux * month * season * year) on the porpoise click detection positive rate per minute (DPRM), number of porpoises click trains per minute, porpoise buzz DPRM and number of porpoises buzz per minute in BJ site at night.

| Source | Dependent Variable | Type III Sum of Squares | df | Mean Square | F | Sig. |
| --- | --- | --- | --- | --- | --- | --- |
| Corrected Model | Click DPRM | 43.49a | 49 | 0.89 | 186.31 | 0.000 |
|  | No. click trains/min | 371.72b | 49 | 7.59 | 67.00 | 0.000 |
|  | Buzz DPRM | 17.17c | 49 | 0.35 | 145.05 | 0.000 |
|  | No. buzzes/min | 181.45d | 49 | 3.70 | 55.00 | 0.000 |
| Intercept | Click DPRM | 1.42 | 1 | 1.42 | 297.11 | 0.000 |
|  | No. click trains/min | 13.85 | 1 | 13.85 | 122.30 | 0.000 |
|  | Buzz DPRM | 0.43 | 1 | 0.43 | 176.41 | 0.000 |
|  | No. buzzes/min | 5.23 | 1 | 5.23 | 77.61 | 0.000 |
| Boat DPRM | Click DPRM | 0.08 | 1 | 0.08 | 16.31 | 0.000 |
|  | No. click trains/min | 0.91 | 1 | 0.91 | 8.00 | 0.005 |
|  | Buzz DPRM | 0.02 | 1 | 0.02 | 9.83 | 0.002 |
|  | No. buzzes/min | 0.24 | 1 | 0.24 | 3.59 | 0.058 |
| Water flux | Click DPRM | 1.27 | 1 | 1.27 | 267.48 | 0.000 |
|  | No. click trains/min | 11.39 | 1 | 11.39 | 100.58 | 0.000 |
|  | Buzz DPRM | 0.45 | 1 | 0.45 | 185.85 | 0.000 |
|  | No. buzzes/min | 5.00 | 1 | 5.00 | 74.30 | 0.000 |
| Lunar | Click DPRM | 0.05 | 2 | 0.03 | 5.52 | 0.004 |
|  | No. click trains/min | 1.20 | 2 | 0.60 | 5.32 | 0.005 |
|  | Buzz DPRM | 0.17 | 2 | 0.08 | 35.10 | 0.000 |
|  | No. buzzes/min | 2.92 | 2 | 1.46 | 21.68 | 0.000 |
| Month | Click DPRM | 1.36 | 11 | 0.12 | 25.91 | 0.000 |
|  | No. click trains/min | 20.03 | 11 | 1.82 | 16.08 | 0.000 |
|  | Buzz DPRM | 0.16 | 11 | 0.01 | 6.11 | 0.000 |
|  | No. buzzes/min | 4.05 | 11 | 0.37 | 5.47 | 0.000 |
| Season | Click DPRM | 1.79 | 3 | 0.60 | 125.42 | 0.000 |
|  | No. click trains/min | 23.42 | 3 | 7.81 | 68.94 | 0.000 |
|  | Buzz DPRM | 1.49 | 3 | 0.50 | 205.15 | 0.000 |
|  | No. buzzes/min | 20.65 | 3 | 6.88 | 102.22 | 0.000 |
| Year | Click DPRM | 6.41 | 3 | 2.14 | 448.35 | 0.000 |
|  | No. click trains/min | 56.41 | 3 | 18.80 | 166.07 | 0.000 |
|  | Buzz DPRM | 3.21 | 3 | 1.07 | 443.14 | 0.000 |
|  | No. buzzes/min | 28.42 | 3 | 9.47 | 140.70 | 0.000 |
| Lunar * Season | Click DPRM | 1.60 | 6 | 0.27 | 56.00 | 0.000 |
|  | No. click trains/min | 23.88 | 6 | 3.98 | 35.15 | 0.000 |
|  | Buzz DPRM | 1.09 | 6 | 0.18 | 75.14 | 0.000 |
|  | No. buzzes/min | 17.25 | 6 | 2.87 | 42.69 | 0.000 |
| Season * Year | Click DPRM | 5.95 | 6 | 0.99 | 208.24 | 0.000 |
|  | No. click trains/min | 59.69 | 6 | 9.95 | 87.86 | 0.000 |
|  | Buzz DPRM | 5.55 | 6 | 0.92 | 382.86 | 0.000 |
|  | No. buzzes/min | 63.00 | 6 | 10.50 | 155.94 | 0.000 |
| Lunar * Season * Year | Click DPRM | 3.90 | 16 | 0.24 | 51.20 | 0.000 |
|  | No. click trains/min | 55.31 | 16 | 3.46 | 30.53 | 0.000 |
|  | Buzz DPRM | 3.22 | 16 | 0.20 | 83.21 | 0.000 |
|  | No. buzzes/min | 49.32 | 16 | 3.08 | 45.78 | 0.000 |
| Error | Click DPRM | 1740.72 | 365417 | 0.00 |  |  |
|  | No. click trains/min | 41374.81 | 365417 | 0.11 |  |  |
|  | Buzz DPRM | 882.61 | 365417 | 0.00 |  |  |
|  | No. buzzes/min | 24604.30 | 365417 | 0.07 |  |  |
| Total | Click DPRM | 1793.00 | 365467 |  |  |  |
|  | No. click trains/min | 41825.00 | 365467 |  |  |  |
|  | Buzz DPRM | 902.00 | 365467 |  |  |  |
|  | No. buzzes/min | 24808.00 | 365467 |  |  |  |
| Corrected Total | Click DPRM | 1784.20 | 365466 |  |  |  |
|  | No. click trains/min | 41746.54 | 365466 |  |  |  |
|  | Buzz DPRM | 899.77 | 365466 |  |  |  |
|  | No. buzzes/min | 24785.74 | 365466 |  |  |  |
| a. R Squared = .024 (Adjusted R Squared = .024) | | | | | | |
| b. R Squared = .009 (Adjusted R Squared = .009) | | | | | | |
| c. R Squared = .019 (Adjusted R Squared = .019) | | | | | | |
| d. R Squared = .007 (Adjusted R Squared = .007) | | | | | | |

**Table S8.** Six-way ANOVA of the effects of parameters (boat traffic *water flux * lunar * month * season * year) on the porpoise click detection positive rate per minute (DPRM), number of porpoises click trains per minute, porpoise buzz DPRM and number of porpoises buzz per minute in YZB site at night.

| Source | Dependent Variable | Type III Sum of Squares | df | Mean Square | F | Sig. |
| --- | --- | --- | --- | --- | --- | --- |
| Corrected Model | Click DPRM | 670.04a | 83 | 8.07 | 502.87 | 0.000 |
|  | No. click trains/min | 21231.36b | 83 | 255.80 | 236.89 | 0.000 |
|  | Buzz DPRM | 618.37c | 83 | 7.45 | 704.21 | 0.000 |
|  | No. buzzes/min | 20666.13d | 83 | 248.99 | 249.20 | 0.000 |
| Intercept | Click DPRM | 0.00 | 1 | 0.00 | 0.04 | 0.842 |
|  | No. click trains/min | 103.42 | 1 | 103.42 | 95.78 | 0.000 |
|  | Buzz DPRM | 0.24 | 1 | 0.24 | 22.82 | 0.000 |
|  | No. buzzes/min | 116.38 | 1 | 116.38 | 116.48 | 0.000 |
| Boat DPRM | Click DPRM | 0.11 | 1 | 0.11 | 6.55 | 0.010 |
|  | No. click trains/min | 0.50 | 1 | 0.50 | 0.46 | 0.498 |
|  | Buzz DPRM | 0.02 | 1 | 0.02 | 1.44 | 0.229 |
|  | No. buzzes/min | 0.13 | 1 | 0.13 | 0.13 | 0.722 |
| Water flux | Click DPRM | 1.82 | 1 | 1.82 | 113.22 | 0.000 |
|  | No. click trains/min | 249.09 | 1 | 249.09 | 230.68 | 0.000 |
|  | Buzz DPRM | 2.16 | 1 | 2.16 | 203.88 | 0.000 |
|  | No. buzzes/min | 251.96 | 1 | 251.96 | 252.17 | 0.000 |
| Lunar | Click DPRM | 5.25 | 2 | 2.62 | 163.42 | 0.000 |
|  | No. click trains/min | 227.74 | 2 | 113.87 | 105.45 | 0.000 |
|  | Buzz DPRM | 3.31 | 2 | 1.65 | 156.40 | 0.000 |
|  | No. buzzes/min | 183.68 | 2 | 91.84 | 91.92 | 0.000 |
| Month | Click DPRM | 34.91 | 9 | 3.88 | 241.61 | 0.000 |
|  | No. click trains/min | 1121.26 | 9 | 124.58 | 115.38 | 0.000 |
|  | Buzz DPRM | 35.73 | 9 | 3.97 | 375.21 | 0.000 |
|  | No. buzzes/min | 1220.08 | 9 | 135.56 | 135.68 | 0.000 |
| Season | Click DPRM | 6.17 | 3 | 2.06 | 128.04 | 0.000 |
|  | No. click trains/min | 95.07 | 3 | 31.69 | 29.35 | 0.000 |
|  | Buzz DPRM | 2.80 | 3 | 0.93 | 88.11 | 0.000 |
|  | No. buzzes/min | 59.30 | 3 | 19.77 | 19.78 | 0.000 |
| Year | Click DPRM | 34.77 | 3 | 11.59 | 722.06 | 0.000 |
|  | No. click trains/min | 437.34 | 3 | 145.78 | 135.00 | 0.000 |
|  | Buzz DPRM | 17.05 | 3 | 5.68 | 537.21 | 0.000 |
|  | No. buzzes/min | 333.45 | 3 | 111.15 | 111.24 | 0.000 |
| Month * Lunar | Click DPRM | 22.67 | 16 | 1.42 | 88.26 | 0.000 |
|  | No. click trains/min | 953.72 | 16 | 59.61 | 55.20 | 0.000 |
|  | Buzz DPRM | 26.22 | 16 | 1.64 | 154.89 | 0.000 |
|  | No. buzzes/min | 1006.80 | 16 | 62.92 | 62.98 | 0.000 |
| Season * Year | Click DPRM | 4.01 | 5 | 0.80 | 49.90 | 0.000 |
|  | No. click trains/min | 39.98 | 5 | 8.00 | 7.41 | 0.000 |
|  | Buzz DPRM | 2.62 | 5 | 0.52 | 49.57 | 0.000 |
|  | No. buzzes/min | 23.00 | 5 | 4.60 | 4.60 | 0.000 |
| Month * Year * Lunar | Click DPRM | 176.34 | 41 | 4.30 | 267.92 | 0.000 |
|  | No. click trains/min | 6603.53 | 41 | 161.06 | 149.16 | 0.000 |
|  | Buzz DPRM | 148.20 | 41 | 3.61 | 341.66 | 0.000 |
|  | No. buzzes/min | 5998.14 | 41 | 146.30 | 146.42 | 0.000 |
| Error | Click DPRM | 6585.90 | 410249 | 0.02 |  |  |
|  | No. click trains/min | 442990.68 | 410249 | 1.08 |  |  |
|  | Buzz DPRM | 4340.22 | 410249 | 0.01 |  |  |
|  | No. buzzes/min | 409901.84 | 410249 | 1.00 |  |  |
| Total | Click DPRM | 7389.00 | 410333 |  |  |  |
|  | No. click trains/min | 466243.00 | 410333 |  |  |  |
|  | Buzz DPRM | 5020.00 | 410333 |  |  |  |
|  | No. buzzes/min | 431926.00 | 410333 |  |  |  |
| Corrected Total | Click DPRM | 7255.94 | 410332 |  |  |  |
|  | No. click trains/min | 464222.04 | 410332 |  |  |  |
|  | Buzz DPRM | 4958.59 | 410332 |  |  |  |
|  | No. buzzes/min | 430567.97 | 410332 |  |  |  |
| a. R Squared = .092 (Adjusted R Squared = .092) | | | | | | |
| b. R Squared = .046 (Adjusted R Squared = .046) | | | | | | |
| c. R Squared = .125 (Adjusted R Squared = .125) | | | | | | |
| d. R Squared = .048 (Adjusted R Squared = .048) | | | | | | |
